# CK2 in triple-negative breast cancer: oncogenic signaling networks and emerging CK2 inhibitor-based combination therapies

**DOI:** 10.1007/s10238-026-02256-7

**Published:** 2026-07-20

**Authors:** Navas Shereef Ellyan, Angel Treasa Alex, Usha Yogendra Nayak, Alan Raj, Rahul K

**Affiliations:** 1 Department of Pharmaceutical Biotechnology, Manipal College of Pharmaceutical Sciences, Manipal Academy of Higher Education, Manipal, 576104 Karnataka India; 2 Department of Pharmaceutics, Manipal College of Pharmaceutical Sciences, Manipal Academy of Higher Education, Manipal, 576104 Karnataka India; 3 Nitte (Deemed to be University), NGSM Institute of Pharmaceutical Sciences (NGSMIPS), Department of Pharmaceutics, 575018 Mangalore, India

**Keywords:** CK2, TNBC, Metastasis, Drug resistance, Combination therapy

## Abstract

The aggressive and highly heterogeneous subtype of breast cancer, triple-negative breast cancer (TNBC), has a poor response to conventional treatments and a high propensity for metastasis, causing unfavorable clinical outcomes. In TNBC, casein kinase 2 (CK2), a constitutively active serine/threonine kinase, plays a key role in controlling several oncogenic signaling pathways. Aberrant CK2 signaling promotes increased transcription of oncogenes, proliferative signaling, DNA repair, epigenetic regulation, epithelial-to-mesenchymal transition (EMT), and cancer stem cell maintenance. Additionally, CK2 helps maintain tumor redox homeostasis by regulating the balance of zinc and copper and by stabilizing immunological checkpoint proteins such as PD-L1. Due to its pleiotropic effects, CK2 overactivation promotes treatment resistance across TNBC subtypes and accelerates tumor growth. Pharmacological inhibition of CK2 can disrupt these communication networks, making TNBC cells more susceptible to both traditional and targeted treatments. By concurrently suppressing compensatory survival mechanisms, CK2 inhibitors have demonstrated synergistic anticancer benefits when used with chemotherapy, PI3K/AKT/mTOR inhibitors, PARP inhibitors, and immunotherapies. This study highlights the complex role of aberrant CK2 signaling in TNBC progression and examines the therapeutic promise of CK2 inhibitor-based combination therapies to overcome resistance and improve treatment efficacy. Current preclinical and clinical research on next-generation CK2 inhibitors highlights their potential as a novel therapeutic approach for TNBC.

**Graphical Abstract:**

**Figure:**
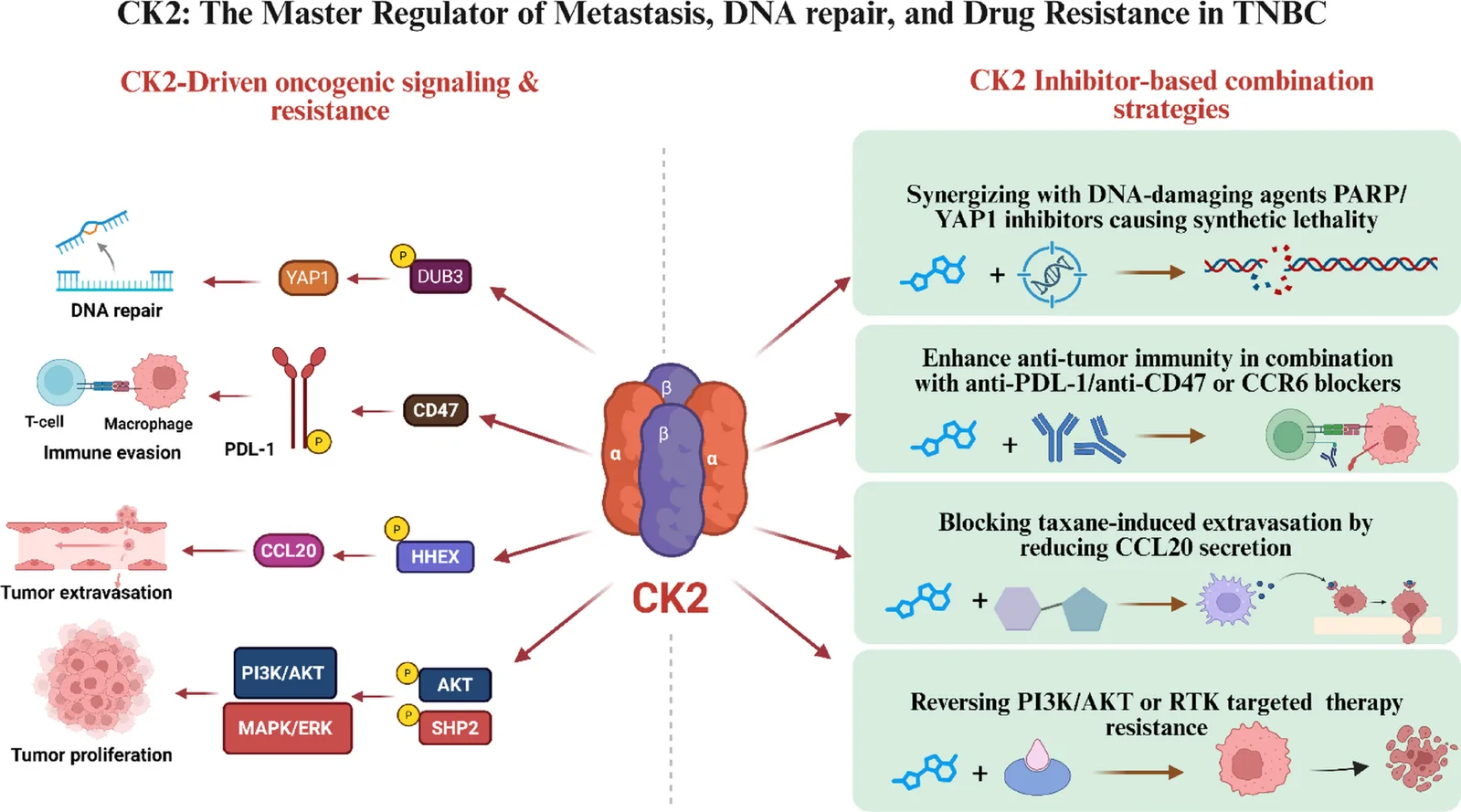

## Introduction

Breast cancer is most common among women around the world, with less than 1% of cases occurring in men. Despite improvements in screening and treatment, about 24% of new cases of breast cancer are triple-negative breast cancer (TNBC), which is one of its subtypes. A major clinical challenge associated with TNBC is the lack of progesterone receptor (PR), human epidermal growth factor receptor 2 (HER2), and estrogen receptor (ER) expression [1]. Additionally, the diagnosis and treatment of TNBC are challenging due to its heterogeneous nature [2]. Conventional treatment options, such as radiation and chemotherapy, often damage healthy cells, while targeted treatments quickly develop resistance [3]. Similarly, certain patient subgroups benefit from immunotherapy and Poly (ADP-Ribose) Polymerase inhibitors (PARPis), but these treatments are limited by toxicity, high cost, and the development of resistance [4–6]. These difficulties underscore the urgent need for combination therapies targeting multiple oncogenic signaling pathways that promote TNBC survival and progression.

TNBCs are frequently characterized by homologous recombination deficiency (HRD), most commonly due to germline or somatic alterations in Breast Cancer Gene 1 and 2 **(**BRCA1/2) and other DNA damage response genes, which predispose them to genomic instability and dependence on compensatory repair pathways [7]. In parallel, TNBC typically exhibits an immunologically active tumor microenvironment (TME) with abundant tumor-infiltrating lymphocytes and checkpoint ligand expression, yet clinical benefit from immunotherapy remains restricted to a subset of patients and is often short-lived [8]. Moreover, TNBC is enriched for mutations in key tumor suppressors such as tumor protein 53 (TP53), contributing to defective cell-cycle control and apoptotic evasion [9]. These convergent molecular features position TNBC as an attractive model for investigating Casein Kinase 2 (CK2)-targeted therapies, because CK2 regulates multiple nodes that sustain DNA repair capacity, inflammatory and immune signaling, and the survival of genomically unstable cells [10]. By simultaneously modulating CK2-dependent DNA repair effectors, chromatin regulators, and cytokine signaling axes, CK2 inhibition can exploit HRD, reshape the immune microenvironment, and enhance the efficacy of PARPi, chemotherapy, and immunotherapies in TNBC [11].

CK2 is a continuously active serine/threonine kinase that plays a key role in regulating various cellular processes vital to the progression of cancer cells [12]. Abnormal CK2 signaling activates several oncogenic pathways, including PI3K/AKT, Nuclear Factor kappa-light-chain-enhancer of activated B cells (NF-κB), Janus Kinase 2 (JAK2)/Signal Transducer and Activator of Transcription 3 (STAT3), and Wingless-related integration site (Wnt)/β-catenin, which contribute to tumor development, metastasis, and resistance to therapy [13]. Phosphorylation of substrates by CK2, such as interleukin-6 (IL-6), Sirtuin 6 (SIRT6), nuclear factor erythroid 2-related factor 1 alpha (Nrf1α), high-mobility group protein A1 (HMGA1), glucose-regulated protein 94 (GRP94/gp96), melanoma cell adhesion molecule (MCAM), and deubiquitinase 3 (DUB3), promotes signaling that supports cell survival and metastasis. Additionally, CK2 promotes DNA repair and epigenetic changes via proteins such as bromodomain-containing protein 4 (BRD4) and histone deacetylases (HDACs). This helps with chromatin remodeling and resistance to therapy. By supporting tumor-initiating cells (T-ICs) and cancer stem cells (CSCs), CK2 also contributes to tumor recurrence and paclitaxel resistance. Furthermore, CK2 regulates zinc and copper ion signaling, which helps keep redox balance. This balance improves metabolic resilience in TNBC cells. Therefore, CK2 activates multiple interconnected oncogenic and survival pathways in TNBC, making it an attractive therapeutic target. Its inhibition has the potential to resensitize tumors to chemotherapy, PARP inhibitors, targeted therapies, and immunotherapies.

### Casein Kinase 2 (CK2)

#### Structure of CK2

**Fig. 1:**
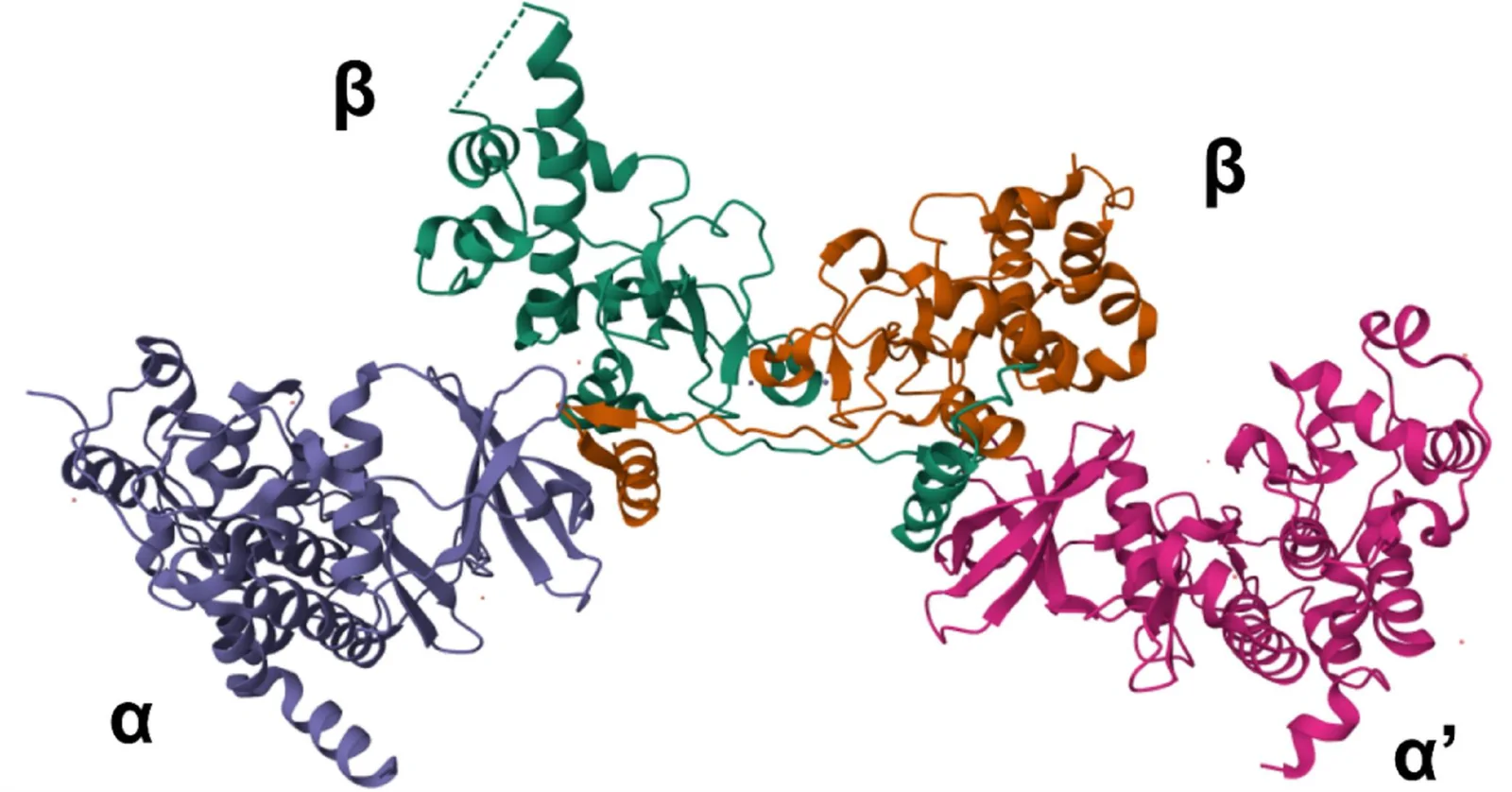
Structure of CK2 (PDB ID: 4DGL)

The holoenzyme CK2 is a highly stable tetrameric complex composed of two catalytic α subunits (CK2α and CK2α′) and two regulatory β subunits (Fig. 1), forming a symmetric α₂β₂ configuration with a dissociation constant (KD) of approximately 4 nM [14]. The human CK2 family is encoded by four genes: CSNK2A1 and CSNK2A2 for the CK2α and CK2α′ catalytic subunits, and CSNK2B for the regulatory CK2β subunit [15]. This architecture enables CK2 to recognize and phosphorylate over 300 substrates, contributing to its role as a major coordinator of intracellular signaling.

The catalytic isoforms CK2α and CK2α’ are structurally similar, but they have different expression patterns and substrate affinities in various tissues. The β subunits have several regulatory roles. They help stabilize the holoenzyme, alter substrate specificity, and influence the catalytic subunit’s activity through an allosteric mechanism [16]. Phosphorylation of Ser209 and Ser53 in CK2α helps stabilize the enzyme and regulate substrate availability. This process influences important functions, such as cell growth, prevention of cell death, and reactions to stress. Moreover, CK2β interacts with other proto-oncogenic kinases, including c-Mos and A-Raf, both members of the MAP kinase kinase kinase (MAPKKK) family, thereby bridging CK2 to Mitogen-Activated Protein Kinase (MAPK)-driven mitogenic signaling [17, 18].

This structural flexibility and interaction capacity make CK2 exceptionally resistant to stress and a central regulator of oncogenic signaling networks. The continuous activity of CK2, largely due to its structurally “open” catalytic site, makes it an attractive target for drug development. Unlike other kinases, CK2 does not require activation by phosphorylation or secondary messengers. These structural features enable small-molecule inhibitors to selectively bind the ATP-binding pocket and suppress downstream signaling.

#### CK2 as a multifunctional protein kinase

CK2 is essential for normal cellular physiology and for disease development. Skeletal morphogenesis, neurogenesis, cardiogenesis, and spermatogenesis are just a few of the developmental processes that depend on the multifunctional CK2 enzyme. Its basic regulatory roles were first identified during early embryogenesis in model organisms such as mice, rats, and chickens. Groucho/Transducin-like enhancer (TLE1) is phosphorylated by CK2 during neuronal development, thereby restricting neuronal differentiation and impeding neural tube formation. Similarly, the absence of the CK2α′ subunit results in mid-gestational death in mice because it is necessary for cardiac morphogenesis. Furthermore, CK2α knockout experiments demonstrated that CK2α deficiency causes oligozoospermia and disrupted spermatogenesis. Moreover, mutations in the CK2β subunit cause skeletal abnormalities and associated lethality, highlighting the critical function of CK2 in endochondral ossification and bone formation [19].

In addition to its role in development, aberrant CK2 signaling causes many illnesses, particularly cancer, where increased CK2 levels lead to carcinogenic transformation. Numerous structural and signaling proteins, transcription factors, and metabolic enzymes are phosphorylated and activated by CK2, thereby integrating cellular pathways into a pro-survival network. Altering transcription factors, such as c-Myc, changes transcriptional activity, which in turn affects metabolic responses, cellular growth, and differentiation. Furthermore, by maintaining phosphorylation-dependent homeostasis, CK2 modulates immune responses and influences skeletal and neurological conditions, such as osteoporosis, osteoarthritis, and Alzheimer’s disease [20].

#### Currently available CK2 inhibitors

The recognition of CK2’s oncogenic role has led to the development of several CK2 inhibitors, including ATP-competitive small molecules, allosteric inhibitors, and peptide-based agents. Among ATP-competitive inhibitors, polyhalogenated benzimidazole/triazole derivatives such as 4,5,6,7-Tetrabromobenzotriazole (TBB) (Ki = 0.4 µM) and its more potent analog, 2-Dimethylamino-4,5,6,7-tetrabromo-1 H-benzimidazole (DMAT) (Ki = 0.04 µM), were among the first selective CK2 inhibitors used as research tools [21]. CX-4945 (Silmitasertib) is an ATP-competitive CK2α inhibitor with a Ki of 0.38 nM. It is orally administered, crosses the blood-brain barrier, has orphan drug status for cholangiocarcinoma, medulloblastoma, and biliary tract cancer, and has advanced to Phase I/II trials [21–23]. CIGB-300 is a Trans-Activator of Transcription (TAT)-peptide inhibitor that disrupts CK2-substrate phosphorylation by targeting the conserved acidic phospho-acceptor domain. It has been tested in Phase I/IIa clinical trials for cervical cancer [24, 25]. Recently, SGC-CK2-1, a next-generation ATP-competitive probe with an IC50 of 2.3 nM for CK2α, was developed as a highly selective chemical probe that surpasses CX-4945 for mechanistic studies [26]. Allosteric and bivalent inhibitors, such as AB668 (Ki = 41 nM), are next-generation agents that bind both the ATP-binding site and the αD allosteric pocket of CK2, inducing cancer-cell-specific death through mechanisms distinct from those of classical ATP-competitive agents [27]. Dual inhibitors targeting CK2 and other oncogenic kinases or epigenetic regulators, such as dual CK2/HDAC inhibitors (e.g., IOR-160) and dual CK2/Proviral Integration Site for Moloney Murine Leukemia Virus 1 (PIM-1) inhibitors derived from DMAT scaffolds, are in preclinical research [28, 29]. The multi-kinase inhibitor ON108600 targets CK2, TNIK, and DYRK1, exemplifying the strategy of exploiting CK2’s overlap with other survival kinases [30, 31].

In this review, we synthesize current evidence on how aberrant CK2 signaling shapes the oncogenic landscape of TNBC, with a particular focus on proliferation, metastasis, chromatin remodeling, DNA damage responses, cancer stem cell maintenance, and immune evasion. We further discuss emerging preclinical and clinical evidence on CK2 inhibitors and highlight rational CK2-based combination strategies to overcome therapeutic resistance and improve outcomes in TNBC.

## Role of CK2 in the progression, metastasis, and treatment resistance of TNBC

Molecular profiling has identified distinct TNBC subtypes, including basal-like 1 (BL1), basal-like 2 (BL2), mesenchymal (M), and luminal androgen receptor (LAR). This highlights the significant heterogeneity within TNBC and helps explain differences in clinical outcomes and treatment responses [32–34]. TNBC is commonly associated with mutations in the BRCA1/2 genes and TP53, leading to genomic instability that contributes to both intrinsic and acquired resistance. Key oncogenic pathways, including the PI3K-AKT-mTOR, Epidermal Growth Factor Receptor (EGFR)/MAPK, and NF-κB pathways, are essential for tumor cell survival, proliferation, and drug resistance [35, 36]. Additional factors, such as overexpression of ATP-binding cassette (ABC) transporters, alterations in the TME, and epithelial-to-mesenchymal transition (EMT), further enhance the ability to evade therapy [37]. Although PARPis are initially effective against TNBC, resistance often develops. This occurs through the activation of EMT transcription factors such as SNAI1, TWIST1, and ZEB1; the loss of Phosphatase and Tensin Homolog on chromosome 10 (PTEN); and adaptive responses driven by the TME [38–40].

CK2 functions as a key amplifier of oncogenic signaling in this complex condition, worsening prognosis and promoting chemoresistance in TNBC. Transcriptomic studies, including Oncomine datasets, reveal substantial overexpression of CK2α and CK2β in breast cancers, particularly in the aggressive TNBC subtype [41, 42]. Table 1 summarizes the roles of CK2 in diverse signaling pathways and their therapeutic implications. In addition to increasing tumor growth and metastatic potential, this multi-pathway engagement creates a dense network of redundant escape routes that underlie resistance to immunotherapy, chemotherapy, and PARP inhibition [43–45]. The sections below describe how CK2-driven pathways contribute to TNBC progression, metastasis, and treatment resistance, and provide an overview of emerging evidence that combining CK2 inhibitors with chemotherapy, PARPi, PI3K/AKT/mTOR inhibitors, immunotherapies, and other pathway inhibitors can yield synergistic antitumor effects.

**Table 1:** Role of CK2 on various signaling pathways, along with therapeutic relevance.

Pathway Axis	Key Mechanism	Therapeutic Implication	Evidence Level	References
CK2–NF-κB / IL-6 axis	CK2 activates the NF-κB/IL-6 signaling axis by phosphorylating IκB-α and upregulating IκB Kinase Epsilon (IKKε), promoting NF-κB nuclear translocation, IL-6 expression, and subsequent STAT3 activation through CK2α and CK2α′-dependent mechanisms.	CK2 inhibitor + proteasome inhibitors (Bortezomib) synergize via ER stress apoptosis	Preclinical (in vitro, in vivo, xenograft) with limited early clinical evidence (Phase I biomarker study)	[46–49]
CK2–PI3K/AKT & MAPK/ERK axis	Amplifies AKT in PTEN-deficient TNBC; SHP2 interaction preserves RTK signaling during EGFR/VEGFR inhibition	CK2 inhibitor + PI3K/AKT/mTOR, RTK (erlotinib, cabozantinib), or MEK inhibitors (trametinib) overcome bypass resistance	Preclinical (in vitro, xenograft, and genetically engineered mouse models)	[50, 51]
CK2-Nrf1α-MRTF-A	Inhibits Nrf1α to derepress MRTF-A, driving ECM stiffening and EMT	CK2 inhibitor + microtubule agents (paclitaxel) or MRTF-A/Rho inhibitors improves penetration and reduces metastasis	Preclinical evidence (cell-based genetic and biochemical studies)	[52–54]
CK2-HMGA1-RAGE	Enables eHMGA1 secretion for paracrine pERK and stromal invasion	CK2 inhibitor + MEK inhibitors (trametinib) or RAGE antagonists disrupt motility and crosstalk	Preclinical (in vitro + in vivo + human tissue validation)	[55]
MCAM-CK2-STAT3	MCAM boosts CK2 for STAT3 activation, promoting plasticity and resistance	CK2 inhibitor + JAK/STAT3 (ruxolitinib) or MCAM blockade reverses aggressive phenotypes	High preclinical evidence (cell lines, patient-derived organoids, transcriptomic analyses, and orthotopic mouse models)	[56]
CK2α-GRP94-Wnt	Stabilizes GRP94-LRP6 for β-catenin signaling and EMT	CK2 inhibitor + Wnt/LRP6 (Mesd) or MEK/PI3K inhibitors restrains lung/brain metastasis	Strong preclinical evidence, supported by CRISPR-mediated genetic manipulation, mechanistic kinase studies, functional metastasis assays, and in vivo xenograft models	[57, 58]
CK2α-HHEX-CCL20	Activates HHEX for CCL20-mediated extravasation; taxane-induced	CK2 inhibitor + taxanes (paclitaxel, docetaxel) or CCR6/NF-κB antagonists prevents relapse	Preclinical translational study comprising in vitro cell-based experiments, animal models, human tissue validation, and bioinformatic analyses	[59]
CK2α-DUB3-YAP1	Enhances DUB3 for YAP1 stabilization, driving DNA repair and EMT	CK2 inhibitor + PARPi, radiation, or YAP-TEAD disruptors (verteporfin) exploit synthetic lethality	Preclinical mechanistic evidence (in vitro, in vivo, biochemical, and clinical tissue correlation studies)	[60, 61]

### CK2-mediated proliferation and metastatic signaling pathways in TNBC

#### CK2-NF-κB axis

**Fig. 2:**
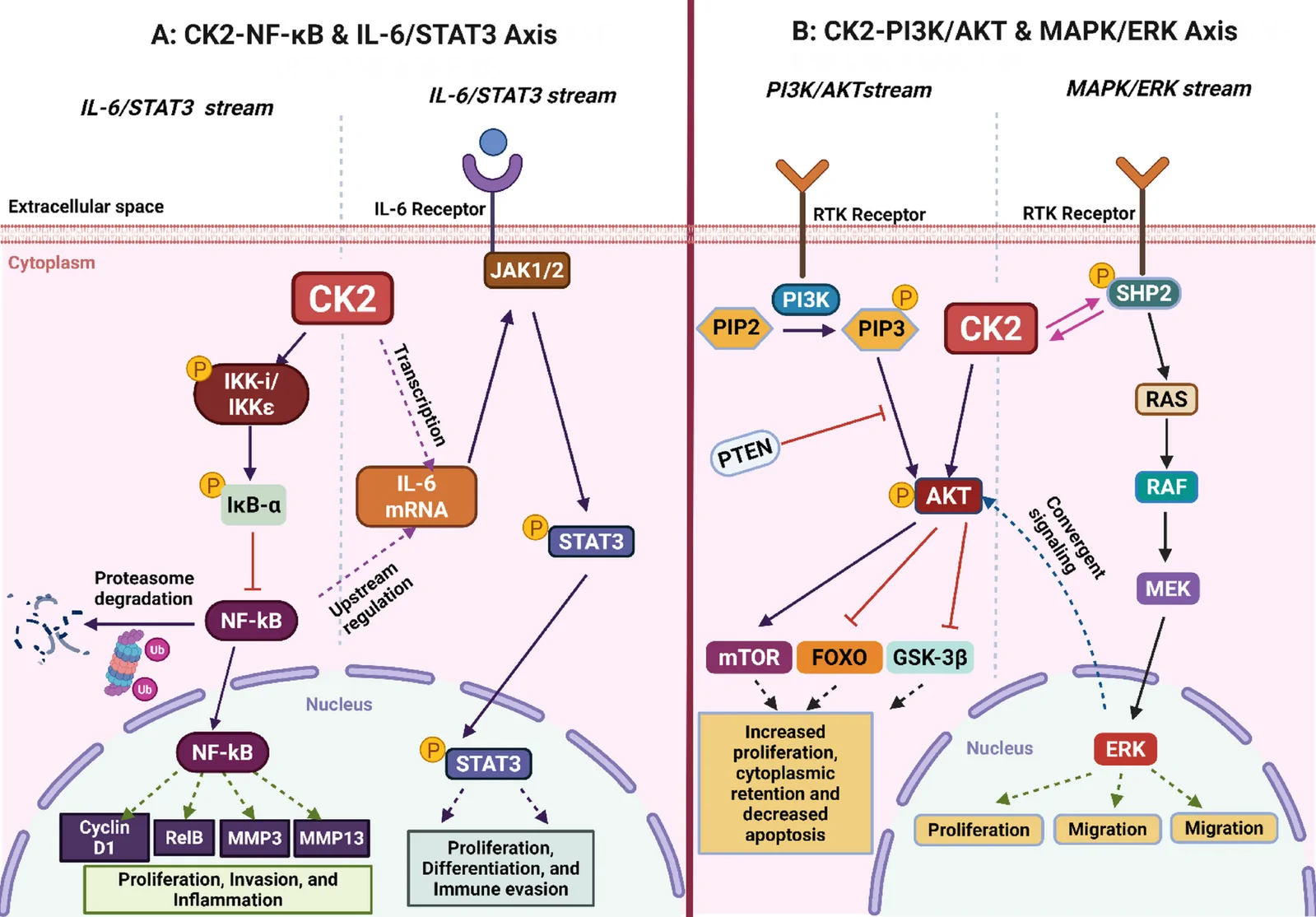
Integration of CK2 signaling axes driving TNBC progression. (**A**) CK2-mediated regulation of the NF-κB and IL-6 signaling axes. CK2 sustains constitutive NF-κB activation by phosphorylating IκB-α within its C-terminal PEST domain, thereby promoting ubiquitin-proteasome-mediated degradation of IκB-α and facilitating NF-κB nuclear translocation. Activated NF-κB induces expression of genes associated with proliferation, invasion, and metastasis, including Cyclin D1, RelB, MMP3, and MMP13. CK2 also maintains IL-6 production by regulating upstream transcription factors, including NF-κB. CK2 promotes STAT3 phosphorylation and activation, thereby regulating genes involved in immune modulation, tumor progression, and metastasis. (**B**) CK2-mediated activation of the PI3K/AKT and MAPK/ERK signaling pathways. In PTEN-deficient settings, CK2 enhances PI3K/AKT signaling by phosphorylating AKT at Ser129, increasing AKT activity beyond that achieved through canonical PDK1- and mTORC2-dependent activation and providing an alternative survival mechanism during PI3K pathway inhibition. Concurrently, CK2 promotes MAPK signaling by activating SHP2, thereby stimulating the RAS-RAF-MEK-ERK cascade and supporting cell proliferation and cell-cycle progression (Created in BioRender. Hariharapura, R. C. (2026) https://biorender.com/euwr60x

NF-κB is a pro-inflammatory transcription factor that regulates genes involved in inflammation, proliferation, survival, and metastasis, thereby promoting tumor growth and progression. In TNBC, constitutive NF-κB activation drives tumor growth, angiogenesis, invasion, and resistance to therapy [62]. CK2 is essential for maintaining active NF-κB signaling in TNBC by modulating multiple regulatory pathways. Inhibitor of κB alpha (IκB-α) usually keeps NF-κB in the cytoplasm as part of an inactive complex (Fig. 2A). CK2 directly phosphorylates IκB-α in its C-terminal PEST (Proline, Glutamic acid, Serine, and Threonine) domain, accelerating its ubiquitin‑proteasome–mediated degradation and thereby promoting NF-κB release and nuclear translocation. In this activated state, NF-κB induces target genes, such as Cyclin D1, RelB, and matrix metalloproteinases 3 & 13 (MMP3 and MMP13), which support cell survival, proliferation, invasion, and inflammatory signaling, all of which contribute to the aggressive behavior of TNBC [46].

CK2 can also enhance NF-κB activity indirectly by upregulating the non-canonical IκB kinases IKK‑i/IKKε. CK2 activity increases both mRNA and protein levels of IKK‑i/IKKε, which phosphorylate IκB-α at Ser36, triggering its degradation and further releasing NF-κB to translocate into the nucleus [47]. Pharmacological or genetic inhibition of CK2 leads to a reduction in IKK-i/IKKε levels. This enhances the stability of IκB-α and reduces NF-κB signaling, thereby preventing tumorigenesis. Because CK2 supports alternative mechanisms that maintain the activity of this pathway, overexpression of CK2 may lead to resistance to therapies that directly target NF-κB or its downstream targets.

From a therapeutic perspective, CK2 inhibition suppresses NF-κB signaling by stabilizing IκB-α and reducing IKK-i/IKKε activity. However, NF-κB may remain active through mechanisms that are independent of CK2. CX-4945 inhibits CK2-specific phosphorylation of p65 at Ser529 but does not affect Ser536 phosphorylation induced by TNF-α, indicating that canonical NF-κB signaling can continue despite CK2 inhibition [63].

Additionally, the non-canonical NIK/IKKα pathway remains active and contributes to TNBC progression, chemoresistance, and metastasis [64, 65]. Consequently, residual NF-κB activity may persist in supporting cellular survival mechanisms even when CK2 is inhibited. The utilization of CK2 inhibitors in conjunction with various NF-κB-targeting agents, such as the proteasome inhibitor Bortezomib, may mitigate this challenge. Preclinical studies suggest that the combination of CX-4945 and Bortezomib suppresses NF-κB signaling and induces apoptosis by more comprehensively disrupting survival pathways than either agent alone [66, 67].

#### CK2-PI3K/AKT & MAPK-ERK axis

The PI3K/AKT/mTOR and MAPK/Extracellular Signal-Regulated Kinase (ERK) pathways, which link receptor tyrosine kinase (RTK) signaling to proliferation, migration, and resistance, are central survival cascades in TNBC. Activated RTKs convert phosphatidylcholine inositol 4,5-bisphosphate (PIP2) to phosphatidylcholine inositol 3,4,5-trisphosphate (PIP3), which attracts and activates AKT and mTOR. This promotes cell growth, inhibits apoptosis, and supports aberrant differentiation and autophagy. The tumor suppressor PTEN counteracts this pathway by dephosphorylating PIP3 back to PIP2 and thereby restraining AKT activation [68]. In PTEN-deficient TNBC, CK2 acts as a critical amplifier of PI3K/AKT signaling and a mediator of resistance to PI3K pathway inhibitors (Fig. 2B). Through STAT3-dependent transcription, the loss of PTEN raises CK2 expression and enhances PI3K/AKT activity [69].

CK2 phosphorylates AKT at Ser129, thereby enhancing its activity beyond that achieved by the PI3K–Phosphoinositide-Dependent Kinase-1 (PDK1) axis [70]. Mechanistically, Ser129, outside the activation loop and hydrophobic motif, when phosphorylated by CK2, induces a conformational change that increases AKT activity beyond that achieved by PDK1 and mTOR complex 2 (mTORC2), acting as a co-activating modification [71]. Under basal conditions, this cooperative phosphorylation contributes to constitutively elevated AKT activity in PTEN-deficient TNBC [72]. When the PI3K/AKT axis is suppressed, CK2-mediated pSer129 remains as a PI3K-independent route that sustains residual AKT activity, bypassing resistance and reducing the effectiveness of pathway inhibitors in PTEN-deficient TNBC. Combined inhibition of CK2 and PI3K/AKT more effectively suppresses survival signaling and induces apoptosis [50]. While CK2-mediated phosphorylation of AKT at Ser129 is well established in TNBC and other cancer cell lines, supported by mouse xenograft studies, there is a clear lack of direct validation of this specific phosphorylation event in patient-derived TNBC samples. This gap presents a major translational challenge that must be addressed before pAKT-Ser129 can be considered a reliable clinical biomarker or tool for patient selection [71].

Additionally, CK2 can phosphorylate EGFR and its downstream effectors, such as AKT and ERK, even when EGFR is inhibited by Erlotinib. This process keeps pro-survival signaling active. Similarly, CK2 enhances Vascular Endothelial Growth Factor (VEGF) and MET signaling when Cabozantinib is administered. This increases the levels of angiogenic factors, such as Hypoxia-Inducible Factor 1-alpha (HIF-1α), which helps cells survive, migrate, and form new blood vessels. Higher CK2α levels in Cabozantinib-resistant cells facilitate their adaptation to MET blockade, thereby strengthening their escape mechanisms [51]. Pharmacologic inhibition of CK2 has been shown to attenuate AKT Ser129 phosphorylation, downregulate MAPK/ERK signaling, and sensitize tumor cells to PI3K inhibitors, RTK inhibitors, and other cytotoxic agents [48, 50].

In TNBC with intact PTEN, CK2 also cooperates with the MAPK axis by interacting with the protein tyrosine phosphatase SHP2 (Src Homology 2 Domain-Containing Protein Tyrosine Phosphatase 2), an upstream regulator of RAS-RAF-MEK-ERK signaling (Fig. 2B) [43, 48]. Mechanistically, CK2 phosphorylation relieves SHP2 autoinhibition, thereby enhancing its phosphatase activity independently of upstream receptor tyrosine kinase signaling [73, 74]. Activated SHP2 directly promotes RAS activation and facilitates the recruitment of Growth Factor Receptor-Bound Protein 2 (GRB2)-Son of Sevenless Homolog 1 (SOS1) complex, enhancing RAS-GTP loading. This process triggers the RAF/MEK/ERK pathway, leading to the expression of genes that support survival and proliferation, including Cyclin D1, MYC, and BCL-2 [74–76]. This CK2-SHP2 axis provides a second, RTK-independent pathway for MAPK activation, working alongside CK2-mediated ERK phosphorylation and nuclear import, thereby enhancing ERK output beyond RTK signaling alone [77]. Persistent ERK1/2 activity drives cell cycle progression, suppresses apoptosis, and sustains MAPK signaling despite RTK inhibitors such as Erlotinib or Cabozantinib, thereby forming a bypass resistance mechanism [75].

#### CK2-IL-6 axis

IL-6, a pleiotropic pro-inflammatory cytokine, promotes angiogenesis, stemness, metastasis, growth, and survival of cancer cells. Inflammatory breast cancers and IL-6-high TNBC subsets are particularly affected by its overexpression. Upstream transcription factors, such as NF-κB, whose activation is maintained by CK2, strictly regulate IL-6 transcription. This links aberrant CK2 signaling to persistent IL-6 production (Fig. 2A). The catalytic subunits of CK2 regulate the IL-6 axis in a compartmentalized manner: the CK2α subunit primarily controls IL-6 expression, whereas CK2α′ contributes to downstream signaling by facilitating the phosphorylation and activation of STAT3, which in turn regulates genes involved in cell survival, proliferation, differentiation, and immune modulation. Pharmacologic inhibition of CK2 with agents such as CX-4945 significantly reduces IL-6 levels in vitro and in vivo, including in inflammatory breast cancer models and early-phase clinical trials. In these trials, CX-4945 treatment was associated with decreased plasma levels of IL-6 and IL-8 and with attenuation of STAT3 signaling [49]. Therefore, CK2 inhibition specifically targets the most critical inflammatory cytokine pathway that underlies inflammation-associated drug resistance and immune evasion in TNBC by downregulating IL-6 production and IL-6-driven JAK/STAT3 activation. Resistance to IL-6/IL-6R inhibitors (such as Tocilizumab and Siltuximab) often results from compensatory upregulation of parallel cytokine pathways and upstream kinases, many of which rely on CK2.

#### CK2-Nrf1α-MRTF-A axis

**Fig. 3:**
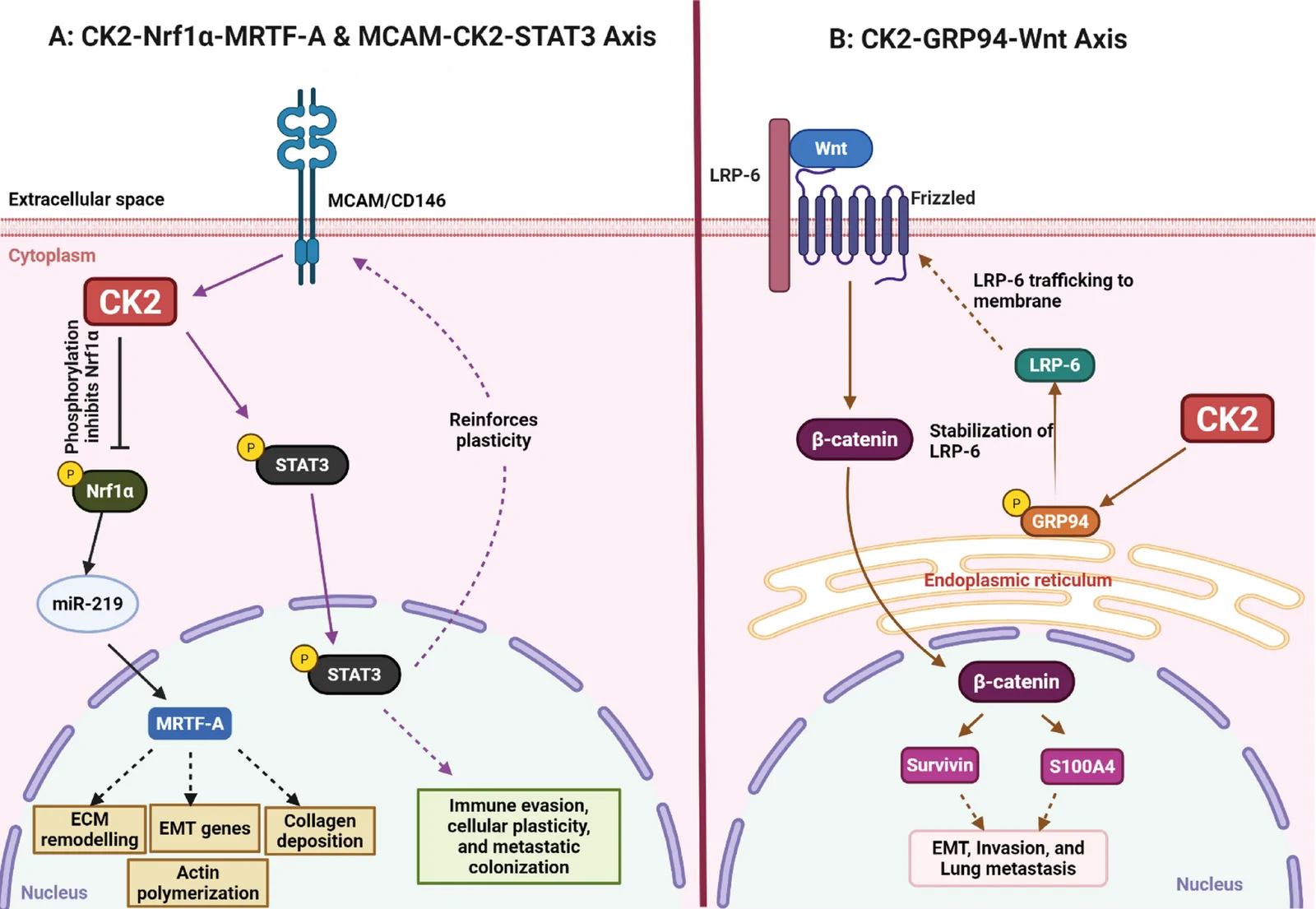
CK2-mediated signaling axes promoting metastasis, extracellular matrix remodeling, and therapy resistance in TNBC. (**A**) Schematic representation of the CK2-Nrf1α-MRTF-A and MCAM-CK2-STAT3 signaling pathways. CK2 directly phosphorylates the tumor-suppressor transcription factor Nrf1α at Ser497, which inhibits its transcriptional activity and stops the expression of miR-219. As a result, MRTF-A accumulates and promotes extracellular matrix (ECM) remodeling. Simultaneously, the cell-surface glycoprotein MCAM enhances CK2’s catalytic activity, leading to STAT3 phosphorylation and activation. Once activated, STAT3 promotes the expression of genes related to cell proliferation, immune evasion, metastatic colonization, and cellular plasticity (**B**) Illustration of the CK2-GRP94-Wnt/β-catenin signaling axis.CK2 phosphorylates the ER chaperone GRP94 at Ser306, stabilizing it and aiding in the maturation and transport of the Wnt co-receptor LRP6 to the cell surface. This process supports the assembly of Wnt receptor complexes and maintains canonical Wnt/β-catenin signaling, thereby increasing the expression of metastasis-related targets such as survivin and S100A4. Activation of this pathway triggers EMT, boosting metastatic spread to distant organs, especially the lungs and brain. (Created in BioRender. Hariharapura, R. C. (2026) https://biorender.com/u7cudki)

Myocardin-Related Transcription Factor A (MRTF-A), a transcriptional co-activator, drives metastatic progression in TNBC by remodeling the extracellular matrix (ECM), promoting collagen deposition, increasing tissue stiffness, and reducing drug penetration [53, 54]. MRTF-A also facilitates actin polymerization, which supports the extravasation, outgrowth, and EMT-associated gene expression of metastatic cells. These activities create a pro-metastatic microenvironment that fosters invasion, therapy resistance, and relapse. Nrf1α, a tumor-suppressive transcription factor, counteracts MRTF-A by indirectly suppressing its activity. Nrf1α transcriptionally upregulates miR-219, a microRNA that targets the 3′ untranslated region (3′-UTR) of MRTF-A mRNA, thereby reducing MRTF-A expression and inhibiting EMT-driven migration and invasion [41]. In TNBC, aberrant CK2 signaling disrupts this protective axis by directly phosphorylating Nrf1α at Ser497, thereby inhibiting its transcriptional activity and miR-219 induction (Fig. 3A) [52]. This CK2-mediated suppression of Nrf1α leads to MRTF-A overexpression, ECM stiffening, enhanced cell motility, and acquired resistance to chemotherapeutics.

Thus, CK2 inhibition is a direct approach to restoring Nrf1α function and disrupting the MRTF-A-driven metastatic program. Agents such as CX-4945 dephosphorylate Nrf1α at Ser497 by inhibiting CK2 activity. This reactivates Nrf1α’s transcriptional output and upregulates miR-219, which suppresses MRTF-A levels [52]. The major difficulties in treating TNBC, such as higher EMT and ECM remodeling, could be mitigated by this mechanism, while improving intratumoral drug delivery. Inhibiting CK2-MRTF-A signaling (with MRTF-A inhibitors or Rho/SRF pathway blockers) could normalize ECM stiffness, enhance chemotherapy penetration, and reduce metastasis. Preclinical evidence supports combining CK2 inhibitors with agents targeting MRTF-A’s downstream effects or other EMT drivers. For instance, CK2 inhibition sensitizes TNBC cells to microtubule-stabilizing antineoplastic agents (e.g., Paclitaxel) by reversing MRTF-A-mediated actin reorganization and migration [53].

#### CK2-HMGA1 axis

**Fig. 4:**
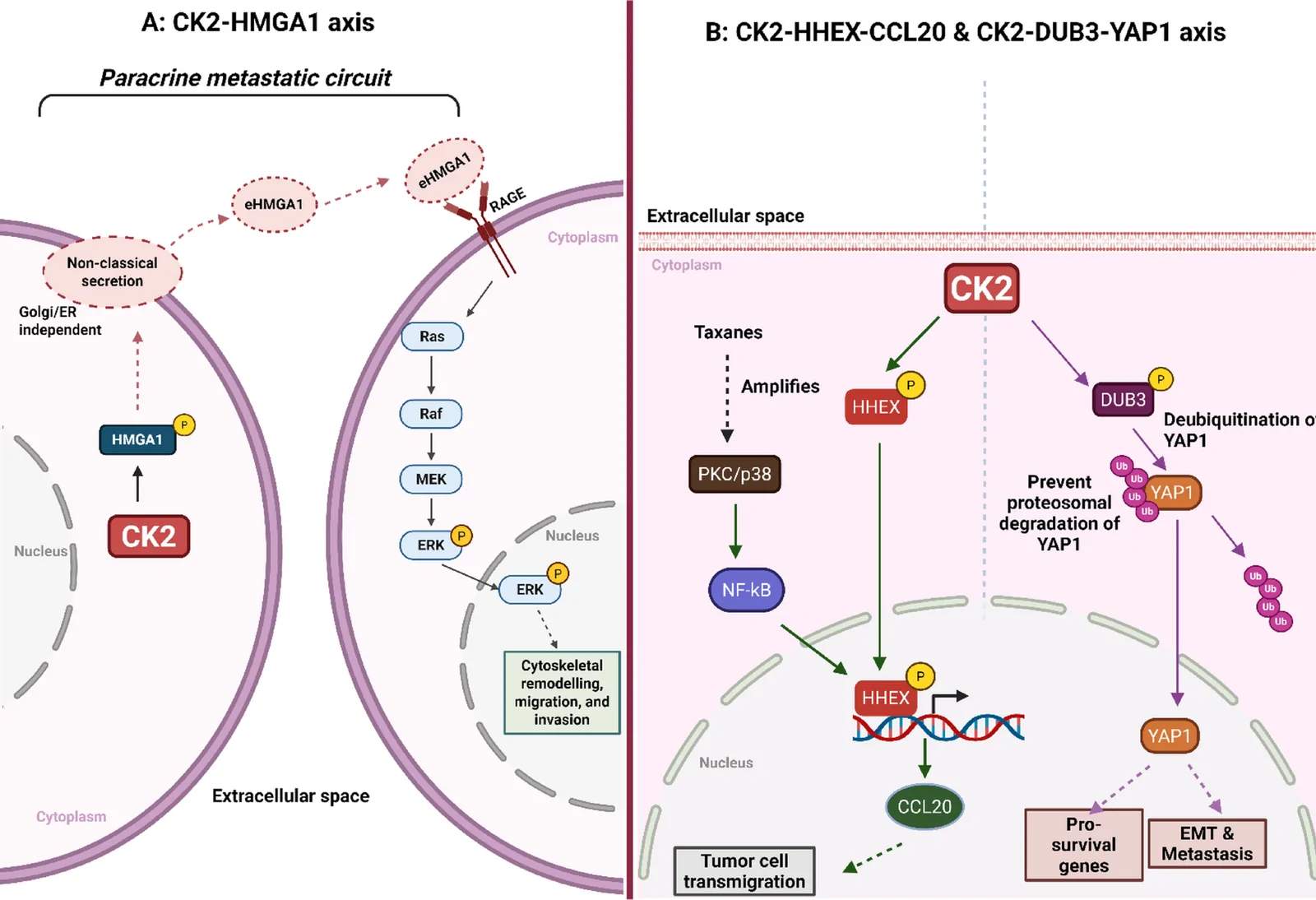
CK2-regulated signaling axes driving metastasis, microenvironmental remodeling, and therapy resistance in TNBC. (**A**) Schematic representation of the CK2-HMGA1-RAGE-pERK paracrine signaling circuit. CK2 phosphorylates the chromatin architectural protein HMGA1, leading to its nonclassical secretion into the tumor microenvironment as extracellular HMGA1 (eHMGA1). Once secreted, HMGA1 interacts with RAGE receptors on nearby tumor and stromal cells, activating MAPK/ERK signaling by phosphorylating ERK. This activation drives cytoskeletal reorganization, reduces cell adhesion, causes matrix detachment, and promotes metastatic spread. (**B**) Illustration of the CK2-HHEX-CCL20 and CK2-DUB3-YAP1 signaling axes. CK2 phosphorylates and activates HHEX, thereby increasing transcription of the chemokine CCL20. This results in increased endothelial permeability, immune cell recruitment, and facilitation of tumor cell extravasation and colonization in distant organs. The prometastatic pathway may be further amplified by taxane chemotherapy, which supports metastatic seeding and the survival of therapy-resistant cancer stem cells. Additionally, CK2α phosphorylates the deubiquitinating enzyme DUB3 at Thr495, enhancing its activity and stabilizing the Hippo pathway effector YAP1 through deubiquitination. The stabilized YAP1 then accumulates in the nucleus, promoting gene expression related to cell survival, metastasis, DNA repair, and therapy resistance (Created in BioRender. Hariharapura, R. C. (2026) https://biorender.com/5quh5s6)

High-mobility group protein A1 (HMGA1), a chromatin architectural regulator, drives TNBC progression through nonclassical secretion. This Golgi/endoplasmic reticulum (ER)-independent mechanism releases proteins via extracellular vesicles or direct translocation across the plasma membrane [55]. CK2 directly phosphorylates HMGA1, enabling its extracellular release as eHMGA1, which then engages the Receptor for Advanced Glycation End-products (RAGE) on neighboring cells and tumor-associated stroma (Fig. 4A). This eHMGA1-RAGE interaction triggers MAPK/ERK signaling activation through phosphorylated ERK (pERK), promoting cytoskeletal remodeling, reduced cell adhesion, enhanced matrix detachment, and increased migration and invasion, all hallmarks of metastatic dissemination in TNBC [55]. Thus, phosphorylated HMGA1 acts as a secreted “metastatic factor,” enhancing pro-invasive signaling in the TME and promoting pre-metastatic niche formation in a paracrine metastatic circuit created by the CK2-HMGA1-RAGE-pERK axis. By continuously producing phosphorylated HMGA1 substrate, aberrant CK2 activity maintains this pathway, contributing to therapy resistance through increased cell motility and stromal reprogramming.

#### MCAM-CK2-STAT3 axis

CD146/MCAM is a transmembrane glycoprotein that functions as a cell adhesion molecule and exhibits increased expression in aggressive TNBC. It serves as a receptor for growth factors and ECM components, promoting tumor growth and the formation of new blood vessels, invasion, and resistance to therapy [78].

In TNBC, the MCAM-CK2-STAT3 axis orchestrates a non-canonical cellular plasticity program that promotes treatment resistance and metastasis, independent of classical EMT markers (Fig. 3A).

MCAM enhances the catalytic activity of CK2, which in turn phosphorylates and activates STAT3, a master transcription factor that upregulates genes supporting cancer cell proliferation, immune evasion, and metastatic colonization. This cooperative signaling creates context-dependent tumor cell states with heightened aggressiveness and adaptive resistance. Elevated MCAM expression is associated with invasive TNBC phenotypes, whereas MCAM deletion in animal models results in less aggressive tumors. MCAM influences the specificity of CK2 substrates in a manner specific to different breast cancer subtypes, enabling adaptable survival strategies that contribute to resistance to chemotherapy and targeted therapies across diverse TNBC groups [56]. Activated STAT3 further reinforces this plasticity, transitioning cells toward pro-metastatic and therapy-refractory states. CK2 inhibition reverses aggressive cellular phenotypes by disrupting MCAM-stimulated CK2 activation, dephosphorylating STAT3, and reducing downstream survival signaling. STAT3 inhibition alone may not be effective to treat resistant TNBC cells, as CK2’s upstream position enables broader pathway activation, including MCAM-dependent inputs. Dual targeting of CK2 and STAT3/JAK (e.g., Ruxolitinib) or MCAM blockade achieves synergistic effects by preventing compensatory reactivation through parallel cytokine and adhesion signaling [56].

#### CK2-GRP94-Wnt axis

GRP94/gp96 is an ER-resident molecular chaperone of the HSP90 family that is strongly induced by tumor-associated stressors such as hypoxia, oxidative stress, and high protein synthesis demand [57, 79]. GRP94 supports tumor growth and metastasis by stabilizing oncogenic proteins, including the Wnt co-receptor Low-Density Lipoprotein Receptor-Related Protein 6 (LRP6), thereby sustaining canonical Wnt/β‑catenin signaling [80]. Elevated GRP94 expression correlates with increased TNBC invasion, brain and lung metastasis, and poor prognosis, highlighting it as a key facilitator of pro-metastatic signaling (Fig. 3B). GRP94 is directly phosphorylated at Ser306 by CK2α, thereby increasing its stability and chaperoning activity for LRP6. Enhanced Wnt ligand-Frizzled-LRP6 complex formation and subsequent β-catenin stabilization are facilitated by phosphorylated GRP94, which more effectively escorts LRP6 from the ER to the cell surface [58]. Enhanced Wnt/β-catenin signaling promotes EMT, invasion, and dissemination of TNBC cells to distant organs, including the lungs, by upregulating metastasis-associated targets, such as survivin and S100A4. Thus, aberrant CK2α-GRP94-LRP6 signaling constitutes a critical axis that couples ER stress adaptation to Wnt-driven metastatic progression.

As CK2α functions in the upstream of the GRP94-LRP6 chaperone-receptor axis, pharmacologic CK2 blockade can reduce GRP94 phosphorylation, destabilize the GRP94-LRP6 complex, disrupt LRP6 trafficking to the plasma membrane, and ultimately hinder Wnt/β-catenin signaling. CK2 inhibitors combined with Wnt/β-catenin or LRP6 pathway inhibitors, such as small-molecule Wnt antagonists or the Mesenchymal-epithelial transition domain-containing protein (Mesd), more effectively suppress metastatic traits by enabling dual inhibition at the chaperone and co-receptor levels.

#### CK2-HHEX-CCL20 axis

In TNBC, CK2α drives tumor cell extravasation, the critical step where circulating cancer cells breach the endothelial barrier to colonize distant organs, through the CK2-HHEX-CCL20 signaling axis (Fig. 4B) [59, 81]. CK2 phosphorylates and activates Hematopoietically Expressed Homeobox (HHEX), a transcription factor that regulates genes involved in inflammation and the immune response. Activated HHEX transcriptionally upregulates C-C Motif Chemokine Ligand 20 (CCL20), an inflammatory chemokine that recruits immune cells, promotes endothelial permeability, and facilitates tumor cell transmigration across vascular barriers [59].CCL20 expression is further amplified by taxane chemotherapy (e.g., Paclitaxel, Docetaxel), which paradoxically enhances breast cancer stem cell self-renewal and survival through PKCζ/p38-mediated NF-κB activation [82]. Thus, the CK2-HHEX-CCL20 axis creates a chemotherapy-induced pro-metastatic niche, in which taxane treatment enhances metastatic seeding efficiency by disrupting endothelial integrity and selecting for resistant stem cells. Due to its dual function in microenvironmental remodeling and intrinsic resistance, CK2 is a key coordinator of post-treatment metastatic relapse.

#### CK2-DUB3-YAP1 axis

Yes-associated protein 1 (YAP1), the principal transcriptional effector of the Hippo pathway, drives tumor growth, EMT, metastasis, and therapeutic resistance in TNBC. Nuclear YAP1 overexpression correlates with high tumor grade, poor prognosis, and reduced response to chemotherapy and radiation [60].

By enhancing repair mechanisms and inhibiting DNA damage responses, YAP1 promotes resistance and enables TNBC cells to survive genotoxic treatments. YAP1 lacks druggable binding pockets, making direct drug inhibition difficult and requiring targeting upstream regulatory mechanisms [60]. Deubiquitinating enzyme 3 (DUB3) stabilizes YAP1 by removing polyubiquitin chains, preventing its proteasomal degradation and sustaining oncogenic activity. DUB3 was previously shown to stabilize SNAI1 and promote TNBC metastasis; it now emerges as a key regulator of YAP1 [40, 61]. CK2α functions as the critical upstream activator, phosphorylating DUB3 at Thr495 to enhance its deubiquitinase activity toward YAP1 [61]. This CK2-DUB3 phosphorylation event creates a stable YAP1 pool, which transcriptionally upregulates pro-survival, pro-metastatic, and DNA repair genes, representing a central axis of TNBC aggressiveness (Fig. 4B) [61].

CK2-DUB3-YAP1 axis provides a druggable target point for YAP1 suppression. CK2 inhibitors prevent YAP1 deubiquitination activity and accelerate YAP1 proteasomal degradation by preventing DUB3 Thr495 phosphorylation. This mechanism helps to counteract YAP1-driven repair and resistance mechanisms, thereby restoring DNA damage accumulation and making cells more vulnerable to radiation and chemotherapy. Preclinical evidence supports testing CK2 inhibitors with PARPi, radiotherapy, chemotherapy, or Hippo/YAP pathway agents, especially in YAP1-high, BRCA-mutant, or homologous recombination (HR)-deficient TNBC, where targeting survival mechanisms may enhance treatment effectiveness.

### CK2 in chromatin remodeling and epigenetics

CK2 serves as a master regulator of chromatin remodeling and epigenetic machinery in TNBC by phosphorylating key epigenetic effectors, including BRD4 and histone deacetylases (HDACs) such as HDAC3 and HDAC6. BRD4, a bromodomain-containing “reader” of acetylated histones, drives TNBC proliferation and metastasis by recruiting transcription factors like c-Myc to promoters of survival genes [34, 42]. By phosphorylating the N-terminal Serine/Proline (NPS) region of BRD4, CK2 functions as a “phospho-switch” that improves transcriptional activation and chromatin binding [42]. Additionally, CK2 facilitates the association of BRD4 with Mediator Complex Subunit 1 (MED1), which contributes to resistance to Bromodomain and Extra-Terminal domain (BET) inhibitors, such as JQ1, by maintaining alternative chromatin interactions [44]. Additionally, CK2-BRD4 complexes with mutant p53 repress p21, enabling uncontrolled proliferation [83].

CK2 enhances HDAC3 activity by phosphorylating Ser424, which in turn promotes chromatin condensation by deacetylating histone tails, leading to tighter nucleosome packing, silencing of tumor suppressor genes, and activation of c-Jun N-terminal kinase (JNK)-NF-κB survival pathways in TNBC [84]. Similarly, CK2 phosphorylates HDAC6 at Ser458, enhancing its deacetylase activity toward glycolytic enzymes, including aldolase, enolase, glyceraldehyde-3-phosphate dehydrogenase, and lactate dehydrogenase, thereby sustaining Warburg metabolism, destabilizing microtubules for motility, and facilitating aggresome-autophagy for proteotoxic stress adaptation in TNBC [45, 85]. Also, a recent study demonstrated that targeting CK2 and HDACs with a dual inhibitor (IOR-160) effectively reduces tumor growth in a TNBC xenograft model [28]. Hence, inhibiting CK2 enhances the efficacy of BET inhibitors by reducing BRD4-dependent signaling and lowering c-Myc levels. It also acts synergistically with HDAC inhibitors to counteract compensatory epigenetic changes.

### CK2 in the DNA repair mechanism

Overexpression of CK2 facilitates resistance to PARPi in BRCA-deficient TNBC by enhancing DNA repair mechanisms, as PARP proteins mediate DNA damage repair, cell cycle regulation, and tumor progression [86]. CK2 phosphorylates and activates PARP1, an important PARP family member that detects DNA damage and repairs it by attaching ADP-ribose molecules to proteins like histones and chromatin remodelers via PARylation [87]. This mechanism enables cancer cells to overcome the effects of chemotherapy, even in the presence of PARPi; upregulated CK2 sustains PARP1 activity, thereby contributing to resistance [88]. Combining PARPi with CK2 inhibitors disrupts these repair mechanisms, creating a synergistic effect that reduces tumor growth and overcomes resistance in BRCA-deficient TNBC. In synthetic lethality-based preclinical TNBC models, the combination of CX-4945 and Olaparib is more effective at inhibiting proliferation and xenograft growth than either monotherapy.

By phosphorylating and activating p53R2, a critical subunit of ribonucleotide reductase (RNR), which is necessary for deoxyribonucleotide (dNTP) synthesis during DNA repair, CK2 plays a crucial role in BRCA-mediated DNA repair through HR. In intact BRCA-proficient cells, CK2-mediated overactivation of p53R2 increases dNTP pools and promotes HR-mediated DNA repair. Overexpression of CK2 leads to a dependence, enabling TNBC cells to repair damage caused by PARPi. Functional BRCA/HR mechanisms typically repair DNA damage caused by PARPi. Inhibiting CK2 impairs HR and induces synthetic lethality in BRCA-proficient TNBC by reducing the availability of p53R2/dNTP. Therefore, CK2i-PARPi combinations (such as Silmitasertib/Olaparib) synergistically reduce proliferation, induce apoptosis, overcome resistance in HR-intact tumors, and shrink xenografts more effectively in TNBC than monotherapy [89].

Chemoresistance in TNBC is also mediated by the CK2-HTATSF1-TOPBP1 signaling axis, in which CK2 phosphorylates and stabilizes HIV-1 Tat-Specific Factor 1 (HTATSF1), a DNA repair protein, allowing it to interact with Topoisomerase II-binding protein 1 (TOPBP1), which is necessary for initiating DNA double-strand break repair via HR. TOPBP1 is upregulated by CK2 overexpression, thereby enhancing DNA repair efficiency and increasing resistance to Talazoparib and Cisplatin. This mechanism may enable TNBC cells to rapidly restore their genomic integrity after chemotherapy. In preclinical TNBC mouse models, inhibiting the CK2-HTATSF1-pTOPBP1 pathway with agents such as Silmitasertib reduces HR and enhances chemotherapy sensitivity. In vivo studies demonstrate that combining CK2 inhibitors with cisplatin results in selective tumor cell death without affecting normal cells [90].

Sirtuins (SIRTs) are NAD-dependent deacetylases that regulate transcription factors, metabolic pathways, and DNA repair mechanisms, thereby significantly promoting metastasis and multidrug resistance in TNBC. Sirtuin 1 (SIRT1) and SIRT6 promote EMT, migration, and invasion by targeting Forkhead box O (FOXO), HIF-1α, and NF-κB, thereby enhancing metastatic behavior via improved mitochondrial function, reduced reactive oxygen species (ROS), and upregulation of efflux transporters/detoxification enzymes [91]. CK2α helps TNBC cells resist chemotherapy-induced DNA damage by phosphorylating SIRT6 at Ser338, activating its DNA repair activity [92]. Targeting the CK2α-SIRT6 axis with inhibitors, such as CX-4945, impairs repair, elevates damage, and sensitizes cells to doxorubicin in preclinical models. CK2 phosphorylates SIRT1 at Ser154, Ser649, Ser651, and Ser683 (mouse equivalents) after ionizing radiation, thereby enhancing SIRT1-mediated DNA repair and radioresistance. CK2 inhibition reduces SIRT1 activity, increasing reactive oxygen species (ROS) and apoptosis, particularly when combined with radiotherapy. Combined CK2–SIRT1 targeting enhances DNA damage, suppresses EMT, and metastasis [93].

### CK2 in cell cycle progression

The retinoblastoma protein 1 (RB1) gene encodes the tumor suppressor retinoblastoma protein (Rb), which regulates cell cycle progression by blocking E2F transcription factors and enforcing the G1/S checkpoint to prevent the replication of damaged DNA [94]. RB1 loss or inactivation, which is frequently observed in TNBC, leads to premature entry into the S phase and replication stress [95, 96]. In cells lacking RB1, compensatory p130 (RBL2) partially preserves control over the G0/G1 phase and acts as the ultimate checkpoint for the cell cycle. CK2 stabilizes p130 by phosphorylating cyclin F, thereby inhibiting its proteasomal degradation. Silmitasertib inhibits CK2, destabilizes p130, thereby eliminating control, inducing premature S-phase entry, replication stress, genomic instability (including micronuclei and aneuploidy), and mitotic cell death via synthetic lethality. In RB1-deficient TNBC xenografts/organoids, CK2 inhibitors show synergism with carboplatin, gemcitabine, or PARPi, enhancing DNA damage, apoptosis, and tumor regression beyond monotherapy. This supports clinically transferable regimens by utilizing the RB1-loss vulnerability in 20–40% of TNBC patients [97].

CIGB-300, a peptide inhibitor of CK2, facilitates cell cycle arrest and apoptosis by inhibiting CK2 regulation of nucleolar phosphoproteins essential for ribosome biogenesis and cell cycle control. In MDA-MB-231 TNBC cells, CIGB-300 increases the sub-G0 apoptotic phase and induces either S-phase or G0/G1 arrest, depending on the cell type, thereby reducing proliferation and clonogenicity [98]. Rac-6 inhibits the phosphorylation and degradation of tumor suppressors p21 and p27 by CK2, thereby restoring their Cyclin-dependent kinase (CDK) inhibition to block the G1-to-S transition, decreasing proliferation, and promoting autophagic cell death in TNBC [29]. By disrupting the nucleolus, CIGB-300 and Paclitaxel synergistically enhance cell cycle arrest at Gap 2/Mitosis Phase (G2/M), promote apoptosis, and reduce metastasis in TNBC models compared with monotherapy. The combination of CK2 inhibitors and chemotherapy targets specific proliferation dependencies, leading to better outcomes in resistant MDA-MB-231 xenograft models and supporting its progression toward clinical applications [98].

CK2β knockdown in MDA-MB-231 TNBC cells causes a G2/M-phase arrest, increased ROS, and apoptosis by raising the levels of markers Bcl-2 Associated X protein (BAX), BCL-XL, and Caspase-3, along with autophagy proteins Beclin-1 and Microtubule-Associated Protein 1 Light Chain 3 Form I (LC3-I). This disrupts the proliferation dependencies that are vital for aggressive TNBC growth. Silencing CK2β promotes EMT and metastasis by upregulating E-cadherin and downregulating invasion/migration markers, including β1-integrin, MMP-9, vimentin, and β-catenin. Upregulation of p21 cell cycle inhibitors further enforces G0/G1 arrest, reducing the possibility of metastasis. CK2 inhibitors that target CK2β, when combined with Taxanes or Doxorubicin, improve ROS/apoptosis, block EMT, and reduce xenograft growth in TNBC models more significantly than monotherapy [99].

CDK11, a critical target in TNBC, controls RNA transcription, cell cycle progression, and proliferation [100]. Studies show a mutual influence where CK2 stabilizes CDK11 pathways, and vice versa. By downregulating CDK11 and/or CK2, Small Interfering RNA (siRNA)-loaded Tenfibgen nanocapsules enable targeted delivery to TNBC cells, thereby significantly inhibiting MDA-MB-231 xenograft growth through transcript cleavage and decreased protein production. Dual targeting produced tumor suppression comparable to that of single treatments without causing additive cell death. When paired with CDK4/6 inhibitors (Palbociclib/Ribociclib) or next-generation CDK2 inhibitors, CK2 inhibitors in TNBC patient-derived xenografts (PDX) models show greater G1/S blockade, over 70% tumor growth reduction, and xenograft regression than either treatment alone. Clinical validation is necessary to confirm the therapeutic benefit of this combination in resistant TNBC, as these synergistic effects depend on the interplay between CK2 and CDK11 [101].

### CK2 maintains T-ICs and CSCs, promoting metastasis and resistance

CK2 plays a vital role in maintaining and promoting the self-renewal of T-ICs, key drivers of TNBC relapse and chemotherapy resistance, which exhibit a Cluster of Differentiation 44 (CD44)-high/CD24-low stem-like phenotype and show enhanced survival against Paclitaxel. CK2 promotes stem-like traits, immune evasion via modulation of myeloid-derived suppressor cells/tumor-associated macrophages, and TME influence, enabling tumor repopulation and metastasis [102]. CK2 has a significant impact on CSC characteristics, such as self-renewal, spheroid formation, and the expression of markers like Aldehyde Dehydrogenase 1 (ALDH1) and Kruppel-Like Factor 4 (KLF4), which contribute to resistance to chemotherapy and radiation and ultimately lead to treatment failure [103]. CK2 inhibitors prevent mammosphere formation, induce G2/M cell cycle arrest and apoptosis in TNBC tumor-initiating cells, and, when combined with Paclitaxel, overcome resistance in MDA-MB-231 xenografts and metastases. Targeting CK2 selectively eliminates breast cancer stem cells, thereby improving chemotherapy efficacy and preventing tumor relapse, advancing clinical treatments in TNBC.

In addition to ALDH1 and KLF4, CK2 influences the pluripotency factors SOX2, OCT4, and NANOG, which support CSC self-renewal, stemness, and chemoresistance in TNBC [104]. SOX2 enhances mammosphere formation, drug efflux, and Paclitaxel resistance, whereas OCT4 and NANOG facilitate EMT, invasion, and a worse prognosis [105–107]. CK2 supports this network primarily through Wnt/β-catenin signaling, in which CK2α stabilizes β-catenin, thereby inducing OCT4 and NANOG expression. Silencing CK2α decreases both factors and hampers the maintenance of tumor-initiating cells [108, 109]. CK2 also enhances β-catenin activity by phosphorylating AKT at Ser129 and facilitates SOX2 expression through STAT3 signaling in TNBC CSCs [109–111].

The multi-kinase inhibitor ON108600 targets CK2, Traf2- and Nck-interacting kinase (TNIK), which modulates the actin cytoskeleton and NF-κB via Traf2/Nck, and Dual-specificity Tyrosine-phosphorylation Regulated Kinase 1 (DYRK1) (a regulator of protein phosphorylation involved in cell cycle, apoptosis, and differentiation) to overcome chemoresistance [112, 113]. ON108600 inhibits T-IC self-renewal by reducing mammosphere/colony formation and stemness markers in TNBC models, while disrupting CK2-regulated DNA repair, which is essential for survival after chemotherapy-induced damage [30, 31]. In TNBC PDX that is resistant to Paclitaxel, ON108600/Paclitaxel combinations show promise for clinical use beyond single-agent therapy by promoting regression, inducing G2/M phase arrest, and eliminating existing metastases with low toxicity.

### CK2 induces immunosuppression and resistance to immune check point inhibitors

**Fig. 5:**
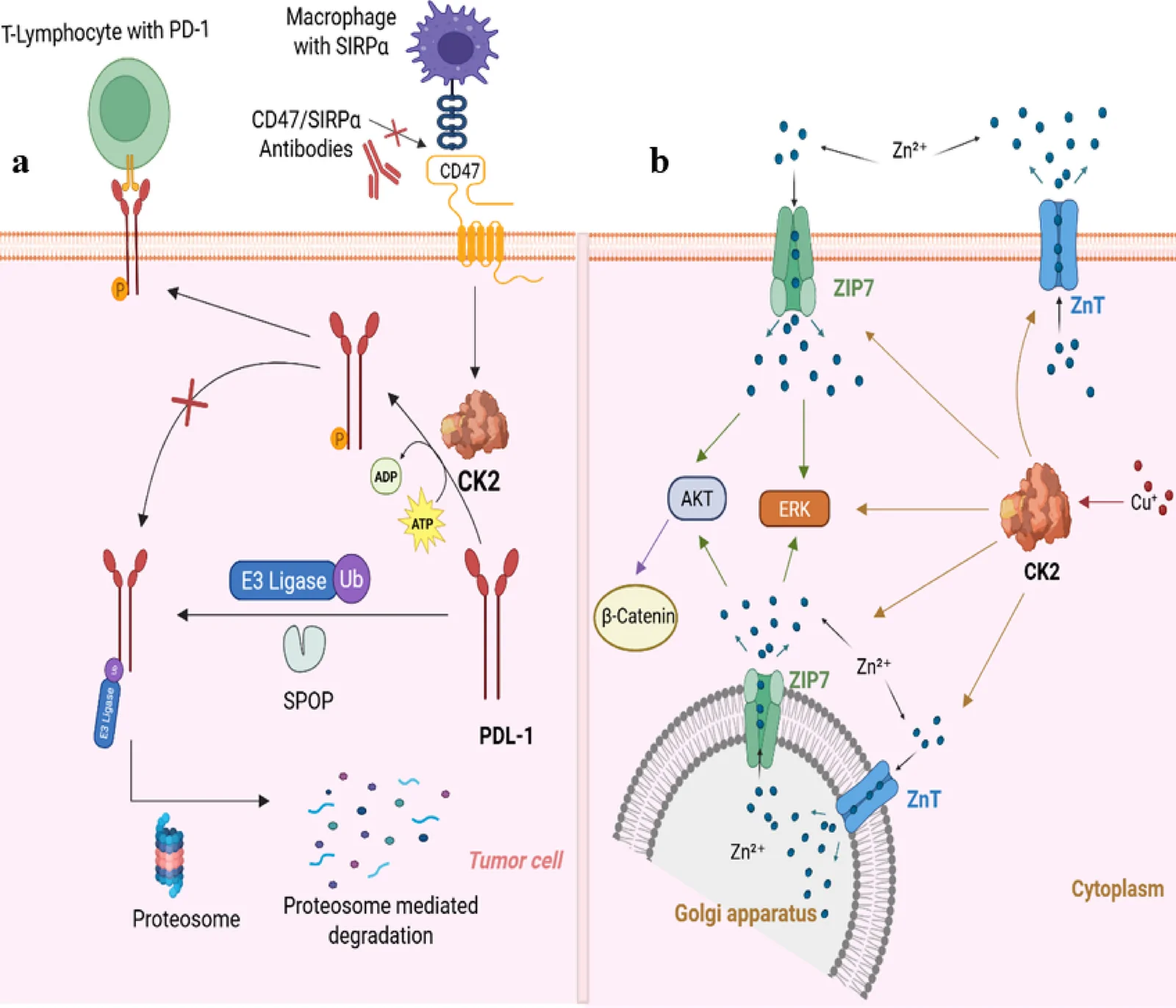
CK2-mediated regulation of immune evasion, ferroptosis, and metal ion homeostasis in TNBC. (**a**) Schematic representation of CK2-driven immune evasion and resistance signaling pathways. CK2 promotes immune escape by phosphorylating PD-L1 at Thr285 and Thr290, preventing its recognition by SPOP and subsequent degradation through the E3 ubiquitin ligase complex. Stabilized PD-L1 accumulates on the tumor cell surface, suppressing T-cell activity through interaction with PD-1 and inhibiting CD47-mediated T-cell priming by dendritic cells. Although CD47 normally protects tumor cells from macrophage-mediated phagocytosis by binding to SIRPα, anti-CD47 therapies can paradoxically increase CK2 expression, thereby contributing to tumor aggressiveness and therapeutic resistance. (**b**) Illustration of the role of CK2 in zinc and copper homeostasis and oncogenic signaling. CK2 phosphorylates and activates the endoplasmic reticulum zinc transporter ZIP7, promoting intracellular zinc release and activating AKT and ERK1/2 signaling pathways that support cell proliferation, survival, and tumor progression. Simultaneously, cuprous copper directly binds to the CK2α catalytic subunit, enhancing its kinase activity and strengthening downstream oncogenic signaling. Copper-mediated CK2 activation increases AKT Ser129 phosphorylation, stabilizes β-catenin, and elevates ERK activity, thereby linking metal ion homeostasis to tumor growth, metastatic potential, and treatment resistance (Created in BioRender. Hariharapura, R. C. (2026) https://BioRender.com/r1immay)

CK2 facilitates immune evasion in the TME by stabilizing programmed death-ligand 1 (PD-L1/CD274), a transmembrane protein expressed on tumor cells, dendritic cells, and macrophages that inhibits T-cell activity, induces exhaustion, and promotes immune escape. CK2 phosphorylates PD-L1 at Thr285 and Thr290 (Fig. 5a). This action prevents PD-L1 from binding to Speckle-type POZ protein (SPOP) and from being degraded by the CUL3 ubiquitin ligase. As a result, surface levels of PD-L1 increase, which suppresses T-cell activation through PD-1 binding. The PD-L1 stabilized by CK2 on dendritic cells blocks CD80. This hinders T-cell priming and provides resistance to immune checkpoint inhibitors (ICIs). CK2 inhibitors, like Silmitasertib, lower PD-L1 levels by promoting the degradation of SPOP and CUL3. They reactivate CD80 on dendritic cells, increase T-cell activity, and reduce PMN-MDSCs and TAMs in syngeneic models [114–116]. Combinations of anti-PD-1, anti-CTLA-4, or anti-TIM-3 antibodies result in tumor regression (62.5% partial/complete) and longer survival. They also show synergy beyond monotherapy.

An emerging aspect of CK2-mediated immune resistance is its influence on ferroptosis, an iron-dependent form of cell death that has gained prominence in immunotherapy-resistant cancers such as TNBC. CK2 inhibits ferroptosis, an iron- and lipid peroxidation-driven death process increasingly linked to TNBC resistance, mainly by phosphorylating Progesterone Receptor Membrane Component 1 (PGRMC1) via the PI3K-AKT pathway. PGRMC1 binds free iron inside cells, reducing the labile iron pool needed for lipid peroxidation and ferroptosis. Notably, PGRMC1 is overexpressed in TNBC, and silencing it makes TNBC cells more sensitive to the ferroptosis inducer Erastin. Conversely, overexpression of PGRMC1 confers resistance, which can be reversed with the antagonist AG205 [117]. This suppressive role is compounded at the mutational level. Oncogenic changes in CK2 substrates, such as TP53, KEAP1, EGFR, Kirsten Rat Sarcoma Viral Oncogene Homolog (KRAS, and Isocitrate Dehydrogenase 1 (IDH1, interfere with the GPX4/Solute Carrier Family 7 Member 11 (SLC7A11 ferroptosis pathway and alter the TME. This disruption reduces ferroptotic cell death and diminishes antitumor immune responses, resulting in resistance to immunotherapy. Ferroptosis inducers can increase PD-L1 levels, while CK2 inhibitors facilitate PD-L1 degradation through the Speckle-Type POZ Protein (SPOP/Cullin 3 (CUL3 ubiquitin ligase pathway. Therefore, combining CK2 inhibitors with ferroptosis-inducing agents could boost tumor cell death and decrease T-cell suppression [118].

Cluster of Differentiation 47(CD47) is an essential immune checkpoint found on TNBC cells. It binds to macrophage Signal Regulatory Protein α (SIRPα), blocking phagocytosis and protecting cancer stem cells, thereby helping them survive (Fig. 5a). Antibodies such as B6H12, CC90002, and SIRPα-Fc target this interaction to help clear tumors via the immune system [103, 115]. However, treatment with anti-CD47 surprisingly increases cancer stemness in MDA-MB-231 cells by elevating CK2 levels, EMT markers such as SNAI1, and the stemness factor ALDH1, thereby promoting resistance. This reduces treatment effectiveness by promoting tumor regrowth. Pre-sensitizing with CK2 inhibitors eliminates CK2-driven stemness and EMT, restoring sensitivity to anti-CD47. It also reduces ALDH1/CD44high populations and enhances phagocytosis and apoptosis in TNBC models when combined with other therapies.

### CK2 regulates intracellular zinc and copper signaling

CK2 regulates zinc homeostasis in TNBC by controlling uptake (ZIP transporters), sequestration, and efflux (ZnT transporters) through phosphorylation of transport pathway proteins. CK2 activates ZIP7, enhancing zinc release from ER stores to trigger AKT/ERK1/2 signaling, which is critical for proliferation/survival (Fig. 5b). Increased intracellular zinc levels, driven by upregulated ZIPs, promote DNA replication and repair. By controlling ZnT-mediated efflux and Golgi sequestration, CK2 preserves homeostasis, avoiding toxic buildup and facilitating treatment escape through repair and survival processes. Increased intracellular zinc levels, driven by upregulated ZIPs, promote DNA replication and repair [119]. CK2 inhibitors (TBB and Silmitasertib) and siRNA reduce ZIP phosphorylation/expression, thereby decreasing uptake and intracellular zinc levels, which impairs proliferation. Blocking ZnT function causes free zinc to accumulate in the cytoplasm. This results in increased ROS, mitochondrial damage, protein dysfunction, and apoptosis [120]. Combinations of CK2 inhibitors with DNA-damaging agents like Doxorubicin or Cisplatin increase toxicity in TNBC models.

Copper binds to specific residues on the CK2α catalytic subunit, such as Met153 and His154, to boost CK2 activity in a redox-dependent manner. This increase in activity depends on the cuprous form of copper. CK2 activity is enhanced by higher quantities of copper within cells and decreased by lower levels (Fig. 5b). Chojnowski et al. (2022) demonstrated that the inhibition of CK2 by Silimitasertib can be reversed by copper supplementation, confirming the copper dependency of CK2 [121]. The copper-mediated reactivation of CK2 can be prevented by combining CK2 inhibition and copper chelation. Copper depletion in TNBC initially increases EMT aggressiveness but eventually reduces metastasis. AKT is phosphorylated at Ser129 by copper-activated CK2, which increases β-catenin activity, treatment resistance, and metastatic potential [122]. By blocking this cascade, CK2i-chelation pairs are more effective than monotherapy at inhibiting invasion, proliferation, and xenografts.

Although CK2-regulated metal ion homeostasis is mechanistically distinct from the signaling axes described earlier, it is interconnected with them. CK2 phosphorylates and activates the ER zinc transporter ZIP7, releasing zinc that directly triggers AKT and ERK1/2 phosphorylation [119]. This amplifies the same PI3K/AKT and MAPK/ERK survival output described in Sect. 2.2 and constitutes an additional bypass mechanism that limits the efficacy of PI3K-targeted therapies [50]. The cuprous ion binds to Met153 and His154 in the CK2α catalytic site, increasing kinase activity and promoting CK2-mediated phosphorylation of AKT Ser129, as well as β-catenin stabilization and activation of the Wnt/stemness pathway [121]. Elevated intracellular copper can enhance surface PD-L1 and immune resistance by increasing CK2α activity, which phosphorylates PD-L1 at Thr285/Thr290, thereby preventing degradation [114–116]. Accordingly, CK2-regulated metal ion homeostasis serves as a redox-sensitive amplifier of oncogenic and immunosuppressive networks.

## Clinical gaps and future directions

Clinical assessments of CK2 inhibitors have cautiously supported CK2 as an important co-target for combination therapies. However, several significant barriers have hindered successful translation. Silmitasertib (CX-4945) demonstrated acceptable tolerability in early Phase I trials (NCT01199718, NCT01352325), with no dose-limiting toxicities, leading to a recommended Phase II dose of 1000 mg twice daily. More recently, a Phase I/expansion trial in basal cell carcinoma (NCT03897036) reported disease control in 65–80% of evaluable patients, representing the most encouraging single-agent clinical activity reported to date [123]. Similarly, the CK2 inhibitor CIGB-300 exhibited manageable toxicity in Phase I studies of cervical cancers, primarily causing local adverse effects. The initial trial did not determine a maximum tolerated dose; however, a later chemoradiotherapy combination study (NCT01639625) identified 70 mg as the maximum tolerated dose [25, 124].

However, several limitations have hindered the clinical translation of CK2 inhibitors. A key concern is CX-4945’s off-target activity; as an ATP-competitive inhibitor, it affects multiple kinases beyond CK2, such as DYRK1A, GSK3β, PIM-1, CLK1–3, FLT3, TBK1, CDK1, and HIPK3. This makes it challenging to optimize dosage and determine whether the therapeutic or side effects are specifically due to CK2 inhibition [24]. Toxicity tends to increase in combination regimens. In a Phase Ib/II trial for cholangiocarcinoma combining silmitasertib with gemcitabine and cisplatin (NCT02128282), 66% of patients experienced diarrhea. Grade ≥ 3 diarrhea, neutropenia, and nausea were frequent, leading to about 13% of patients discontinuing treatment. Additionally, two deaths were regarded as possibly related to the treatment [125, 126]. In parallel, the lack of validated predictive biomarkers led to the enrollment of largely unselected patient populations. Unlike oncogenic kinases such as ABL or EGFR, CK2 operates through a non-oncogene-addiction mechanism and lacks recurrent activating mutations that could guide patient selection [23]. Consequently, signs of effectiveness were weaker, and inadequate pharmacodynamic monitoring, combined with underpowered early-phase trial designs, made it difficult to confirm target engagement in specific molecular subgroups [127, 128]. Overall, these limitations suggest that the modest clinical results to date are due to issues in drug design and development rather than the absence of a biological basis for targeting CK2. The existing evidence offers cautious optimism for CK2 inhibitors, especially when used as part of well-planned combination therapies. However, there is still no clinical data on their safety or effectiveness in TNBC.

Rational combination regimens provide a crucial advantage in addressing these challenges. Table 2 examines various combination regimens demonstrating promising outcomes in both preclinical and clinical settings. Furthermore, the table offers proof of concept for additional potential combination strategies to be investigated in the future. Additionally, tumor-targeted formulations offer another way to overcome these challenges. Innovative delivery methods, such as nanoparticles, liposomes, or ligand-conjugated carriers, enhance tumor-specific uptake via passive mechanisms (such as the EPR effect) and active targeting. This results in higher localized drug concentrations within the tumor and lowers systemic side effects [129, 130]. This minimizes off-target toxicity and improves the therapeutic index, which is especially important for a ubiquitously expressed kinase such as CK2 [131, 132].

**Table 2:** Emerging Scope for CK2 Inhibitor-Based Combination Treatment Approaches

Therapeutic Category	Combination Partners	Mechanistic Rationale & Evidence Level for Combination Strategies	References
Chemotherapy	Taxanes (Paclitaxel, Docetaxel)	CK2 inhibition eliminates taxane-resistant T-ICs by blocking CCL20 signaling and MRTF-A-mediated actin remodeling, as demonstrated in xenograft and patient-derived xenograft models	[52–54]
	Platinum Agents (Cisplatin, Carboplatin)	Preclinical evidence indicates that CK2 promotes cisplatin resistance through YAP1 stabilization and the HTATSF1-TOPBP1 axis, while CK2 inhibition enhances cisplatin sensitivity and therapeutic efficacy	[90]
	Doxorubicin	In vitro studies and analysis of 142 human breast cancer tissues support combining CK2 inhibition with doxorubicin to target the CK2α-SIRT6 DNA repair pathway and disrupt zinc homeostasis, thereby promoting oxidative stress-mediated cell death	[91, 92]
	Gemcitabine	Based on preclinical data, CK2 inhibition enhances gemcitabine efficacy in RB1-deficient cells by disrupting the CK2-cyclin F-p130 axis and inducing replication stress. Improved progression-free survival in advanced cholangiocarcinoma was demonstrated in early clinical investigations; however, further development was halted due to the issues outlined in Sect. 3	[97]
Targeted Therapy	PARPi (Olaparib, Talazoparib)	Cell lines, human tumor tissue microarrays, and xenograft models provide preclinical evidence that CK2 inhibition impairs DNA repair and destabilizes YAP1, resulting in a BRCA-like deficiency that enhances the effectiveness of PARPi such as olaparib and talazoparib	[89]
	PI3K/AKT inhibitors (GDC0941, AZD8186 & MK2206)	Evidence from cell, genomic, and murine models shows that CK2 inhibition suppresses compensatory EGFR-PI3K/AKT signaling and overcomes adaptive resistance, thereby enhancing the efficacy of AZD8186 and related PI3K/AKT pathway inhibitors	[50]
	RTK inhibitors (Erlotinib, Cabozantinib)	In vitro evidence with strong mechanistic and translational validation shows that CK2 inhibition enhances RTK inhibitor efficacy by suppressing EGFR/c-Met signaling and preventing compensatory PI3K/AKT pathway reactivation associated with drug resistance	[51]
	IL-6/JAK/STAT3 inhibitors	Early clinical validation, along with in vitro and in vivo data, demonstrates that CK2 inhibition disrupts IL-6/JAK/STAT3 signaling, underscoring the potential of combining CK2 and IL-6/JAK/STAT3 inhibitors to improve antitumor efficacy	[49]
	Wnt/LRP6 Path inhibitors	CK2-driven GRP94 stabilization increases LRP6/Wnt/β-catenin signaling and metastasis, as shown by in vitro and in vivo results, providing a foundation for combined CK2 and Wnt/LRP6 suppression	[57, 58]
	CDK 11 inhibitors	In vitro and in vivo evidence identify CK2 and CDK11 as interconnected survival kinases in TNBC; although synergistic effects were not observed, their reciprocal regulation justifies combined targeting	[101]
	Hippo pathway activators	The use of CK2 inhibitors in conjunction with YAP1-targeted therapeutics is validated by in vitro and in vivo evidence that the CK2-DUB3-YAP1 axis drives tumor growth and chemotherapy resistance	[60, 61]
Immunotherapy	ICIs (Anti-PD-1, CTLA-4, TIM-3)	Translational immune-mechanistic validation by in vitro and in vivo studies demonstrates that CK2 inhibition enhances antitumor immunity and, when paired with anti-PD-1, CTLA-4, or TIM-3 blockade, improves antitumor efficacy and survival, underscoring the potential of combined CK2 and immune checkpoint inhibition	[114, 116]
	Anti-CD47 antibodies	Targeting CK2α-CD47/SIRPα can induce cancer stemness and EMT in TNBC cells, as evidenced by in vitro mechanistic and transcriptomic studies. This justifies combining CK2 inhibitors with CD47/SIRPα-directed therapies to reduce protumor signals and overcome resistance	[103]
	CCR6 blockers/anti-inflammatories	In vitro mechanistic and transcriptomic evidence shows that SIRPα-Fc-mediated CD47 targeting can induce CK2α expression, cancer stemness, and EMT in TNBC cells, establishing a basis for combining CK2 inhibitors with CD47/SIRPα therapies to reduce protumor signaling and resistance	[59, 81]
Epigenetic Therapy	BET inhibitors (JQ1, OTX015)	CK2 phosphorylation of BRD4 promotes stability and confers BET inhibitor resistance, as demonstrated by in vitro and TNBC xenograft studies. Tumor development caused by c-MYC, mutant p53, and CXCR4 is all reduced by BET inhibition. This suggests that CK2 inhibitors enhance the efficacy of BET inhibitors and overcome resistance	[44, 83]
	HDAC inhibitors.	Combining CK2 and HDAC inhibitors as a TNBC treatment is supported by biochemical, mechanistic, structural, and TNBC xenograft studies. HDAC-mediated epigenetic regulation, particularly CK2-dependent HDAC6 activation, is linked to CK2-driven carcinogenic signaling. This dual targeting has significant anticancer effects in vivo by suppressing AKT signaling, increasing α-tubulin acetylation, and impairing tumor survival and stress adaptation	[28, 85]
Others	Copper chelators	Copper increases CK2 activity and can partially restore CK2 signaling following CX-4945 therapy, as shown in vitro, genetic models, and cell-based studies. Combining CK2 inhibitors with copper-chelating drugs may improve antitumor efficacy, reduce resistance, and better suppress the oncogenic CK2/AKT signaling pathway	[121]
	Radiation Therapy	Cellular and molecular research, as well as radiobiological results, support combining radiation therapy with CK2 inhibition. CK2 inhibition increases radiation-induced apoptosis, decreases clonogenic survival, overcomes radioresistance, and improves tumor management by impairing DNA repair and survival signaling, including stress pathways such as SIRT1	[93]

## Conclusion and future perspectives

Protein kinase CK2 is identified as a central regulator of therapeutic resistance, metastasis, and immune evasion in TNBC. Instead of acting merely as a linear oncogenic driver, CK2 functions as a global signaling hub that coordinates growth factor pathways, inflammatory responses, epigenetic flexibility, DNA repair, cell cycle regulation, cancer stem cell traits, and immune checkpoint control. Its upstream role explains why CK2 overexpression leads to widespread resistance to chemotherapy, PARPi, targeted therapies, and immunotherapy, and why treatments targeting downstream components often fail due to rapid reactivation of the pathway. From a translational perspective, inhibiting CK2 marks a shift from targeting individual pathways to a broader network-disruption paradigm. By simultaneously interfering with multiple survival pathways, including PI3K/AKT, NF-κB, Wnt/β-catenin, STAT3, BRD4/HDAC epigenetic mechanisms, and HR pathways, CK2 inhibitors make TNBC cells more susceptible to various therapies. They also help reduce metastasis and prolong the survival of tumor-initiating cells. Notably, preclinical and early clinical data show that CK2 inhibition not only enhances the efficacy of standard treatments such as taxanes, platinum drugs, and PARPi but also restores response to ICIs by reversing CK2-mediated PD-L1 stabilization and altering the immunosuppressive TME.

Although CK2 inhibitors such as silmitasertib (CX-4945) and CIGB-300 have demonstrated encouraging therapeutic potential in preclinical models and early-phase clinical studies, available clinical evidence remains limited. Published data suggest manageable toxicity profiles in some monotherapy settings; however, clinically significant adverse events have also been reported in combination regimens. Therefore, further clinical studies are required to establish their safety, efficacy, and optimal therapeutic application, particularly in TNBC, where clinical data remain lacking. This makes CK2 a suitable co-target rather than a standalone replacement. Effective combination of CK2i-PARPi in tumors with intact DNA repair, CK2i-chemotherapy to prevent therapy-related metastasis, and CK2i-ICI pairings to combat immune resistance. These emerging interventions offer promising options for expanding treatments in biomarker-specific subtypes of TNBC.

Additionally, new insights into CK2-related vulnerabilities, including RB1 loss, MCAM expression, inflammatory cytokines, and metal ion regulation, offer opportunities for patient stratification and the targeted use of CK2-centered therapies. Looking ahead, successful clinical translation will involve incorporating CK2 inhibition into biomarker-guided combination strategies, refining dosing schedules to leverage synthetic lethality while reducing toxicity, and creating next-generation CK2 inhibitors or dual-acting molecules with improved selectivity. Since resistance remains the main challenge in TNBC treatment, targeting CK2 offers a comprehensive approach to address both innate and adaptive escape mechanisms. Overall, these developments position CK2 inhibition not just as another targeted therapy but as a fundamental platform for sustained, multi-faceted treatment in aggressive and resistant TNBC.

## Data Availability

No datasets were generated or analysed during the current study.

## Abbreviations

ABC: ATP-binding cassette
AKT: Protein Kinase B
ALDH1: Aldehyde Dehydrogenase 1
BAX: Bcl-2 Associated X protein
BET: Bromodomain and Extra-Terminal domain
BL1/2: Basal-like1/2
BRD4: Bromodomain-containing protein 4
CCL20: C-C Motif Chemokine Ligand 20
CDK: Cyclin-dependent kinase
CD44: Cluster of Differentiation 44
CD47: Cluster of Differentiation 47
CK2: Casein Kinase 2
CSC: Cancer Stem Cell
dNTP: Deoxyribonucleotide
DUB3: Deubiquitinase 3
DYRK1: Dual-specificity Tyrosine-phosphorylation Regulated Kinase 1
ECM: Extracellular matrix
ER: Estrogen receptor
EGFR: Epidermal Growth Factor Receptor
EMT: Epithelial-to-Mesenchymal Transition
ERK: Extracellular Signal-Regulated Kinase
FOXO: Forkhead box O
G2/M: Gap 2 / Mitosis Phase
GRP94/gp96: Glucose-Regulated Protein 94
HER2: Human epidermal growth factor receptor 2
HDAC: Histone deacetylase
HDAC3: Histone Deacetylase 3
HDAC6: Histone Deacetylase 6
HHEX: Hematopoietically Expressed Homeobox
HIF-1α: Hypoxia-Inducible Factor 1-alpha
HMGA1: High Mobility Group AT-Hook 1
HR: Homologous Recombination
HTATSF1: HIV-1 Tat Specific Factor 1
ICIs: Immune checkpoint inhibitors
IL-6: Interleukin 6
IKKε: IκB Kinase Epsilon
IκB-α: Inhibitor of κB alpha
KLF4: Kruppel-Like Factor 4
LC3- I: Microtubule-Associated Protein 1 Light Chain 3 Form I
LDHA: Lactate Dehydrogenase A
LRP6: Low-Density Lipoprotein Receptor-Related Protein 6
LAR: Luminal androgen receptor
M: Mesenchymal
MAPK: Mitogen-Activated Protein Kinase
MAPKKK: MAP kinase kinase kinase
MCAM: Melanoma Cell Adhesion Molecule
MED1: Mediator Complex Subunit 1
Mesd: Mesenchymal-epithelial transition domain-containing protein
MMP3 & MMP13: Matrix metalloprotease 3 & 13
MRTF-A: Myocardin-Related Transcription Factor A
NF-κB: Nuclear Factor kappa-light-chain-enhancer of activated B cells
NPS: N-terminal Serine/Proline
Nrf1α: Nuclear factor erythroid 2-related factor 1 alpha
PR: Progesterone receptor
PARP1: Poly (ADP-Ribose) Polymerase 1
PARPi: PARP Inhibitor
PDX: Patient-derived xenografts
PD-L1: Programmed Death-Ligand 1
pERK: Phosphorylated ERK
PEST: Proline, Glutamic acid, Serine, and Threonine
PIP2: Phosphatidylcholine inositol 4,5-bisphosphate
PIP3: Phosphatidylcholine inositol 3,4,5-trisphosphate
PI3K: Phosphoinositide 3-Kinase
PTEN: Phosphatase and Tensin Homolog on chromosome 10
RAGE: Receptor for Advanced Glycation End-Products
Rb1: Retinoblastoma Protein 1
RNR: Ribonucleotide reductase
ROS: Reactive oxygen species
RTK: Receptor tyrosine kinase
SHP2: Src Homology 2 domain-containing protein tyrosine phosphatase 2
SIRPα: Signal Regulatory Proteinα
SIRT6: Sirtuin 6
SIRT1: Sirtuin 1
siRNA: Small Interfering RNA
SPOP: Speckle-type POZ protein
STAT3: Signal Transducer and Activator of Transcription 3
T-IC: Tumor-Initiating Cell
TBB: 4,5,6,7-Tetrabromobenzotriazole (A CK2 inhibitor)
TLE1: Groucho/Transducin-like enhancer
TME: Tumor microenvironment
TNBC: Triple-Negative Breast Cancer
TNIK: Traf2- and Nck-Interacting Kinase
TOPBP1: Topoisomerase II-binding protein 1
VEGFR: Vascular Endothelial Growth Factor
Wnt: Wingless-related integration site (Signaling pathway)
YAP1: Yes-Associated Protein 1
ZIP7: Zinc Transporter Protein 7
ZnT: Zinc Transporter
3′-UTR: 3′ untranslated region

## Declarations

### Declaration on the use of artificial intelligence-assisted technologies

The authors declared that the artificial intelligence-assisted tool was used for language editing and grammatical refinement during the preparation of this manuscript.

## References

[1] Houghton SC, Hankinson SE. Cancer Progress and Priorities: Breast Cancer. Cancer Epidemiology. Biomarkers Prev. 2021;30(5):822–44. 10.1158/1055-9965.EPI-20-1193. PMC8104131 33947744

[2] Leon-Ferre RA, Goetz MP. Advances in systemic therapies for triple negative breast cancer. BMJ. 2023;381:e071674. 10.1136/bmj-2022-071674 . PubMed PMID: 37253507. 37253507

[3] Kinnel B, Singh SK, Oprea-Ilies G, Singh R. Targeted Therapy and Mechanisms of Drug Resistance in Breast Cancer. Cancers. 2023;15(4):4. 10.3390/cancers15041320. PMC9954028 36831661

[4] Hanna A, Balko JM. Breast cancer resistance mechanisms: challenges to immunotherapy. Breast Cancer Res Treat. 2021;190(1):5–17. 10.1007/s10549-021-06337-x. PubMed PMID: 34322780; PubMed Central PMCID: PMC8560575. PMC8560575 34322780

[5] Andre F, Filleron T, Kamal M, Mosele F, Arnedos M, Dalenc F, et al. Genomics to select treatment for patients with metastatic breast cancer. Nature. 2022;610(7931):343–8. 10.1038/s41586-022-05068-3. 36071165

[6] Scheidemann ER, Shajahan-Haq AN. Resistance to CDK4/6 Inhibitors in Estrogen Receptor-Positive Breast Cancer. Int J Mol Sci. 2021;22(22):22. 10.3390/ijms222212292. PMC8625090 34830174

[7] Li J, Jia Z, Dong L, Cao H, Huang Y, Xu H, et al. DNA damage response in breast cancer and its significant role in guiding novel precise therapies. Biomark Res. 2024;12(1):111. 10.1186/s40364-024-00653-2. PMC11437670 39334297

[8] Figueroa S, Reyes N, Tiwari RK, Geliebter J. Targeting the Tumor Microenvironment in Triple-Negative Breast Cancer: Emerging Roles of Monoclonal Antibodies and Immune Modulation. Cancers. 2026;18(3):412. 10.3390/cancers18030412. PMC12896577 41681885

[9] El Hejjioui B, Lamrabet S, Amrani Joutei S, Senhaji N, Bouhafa T, Malhouf MA, et al. New Biomarkers and Treatment Advances in Triple-Negative Breast Cancer. Diagnostics. 2023;13(11):1949. 10.3390/diagnostics13111949. PMC10252475 37296801

[10] Firnau MB, Brieger A. CK2 and the Hallmarks of Cancer. Biomedicines. 2022;10(8):1987. 10.3390/biomedicines10081987. PMC9405757 36009534

[11] Chen L, Zhang S, Li Q, Li J, Deng H, Zhang S, et al. Emerging role of Protein Kinase CK2 in Tumor immunity. Front Oncol. 2022;12. 10.3389/fonc.2022.1065027. PMC9752132 36530985

[12] Day-Riley S, West RM, Brear PD, Hyvönen M, Spring DR. CK2 Inhibitors Targeting Inside and Outside the Catalytic Box. Kinases Phosphatases. 2024;2(2):2. 10.3390/kinasesphosphatases2020007.

[13] Ortega CE, Seidner Y, Dominguez I. Mining CK2 in Cancer. PLoS ONE. 2014;9(12):e115609. 10.1371/journal.pone.0115609. PMC4277308 25541719

[14] Lolli G, Ranchio A, Battistutta R. Active Form of the Protein Kinase CK2 α2β2 Holoenzyme Is a Strong Complex with Symmetric Architecture. ACS Chem Biol. 2014;9(2):366–71. 10.1021/cb400771y. 24175891

[15] Trembley JH, Kren BT, Afzal M, Scaria GA, Klein MA, Ahmed K. Protein kinase CK2 – diverse roles in cancer cell biology and therapeutic promise. Mol Cell Biochem. 2023;478(4):899–926. 10.1007/s11010-022-04558-2 . PubMed PMID: 36114992; PubMed Central PMCID: PMC9483426. PMC9483426 36114992

[16] Cesaro L, Zuliani AM, Bosello Travain V, Salvi M. Exploring Protein Kinase CK2 Substrate Recognition and the Dynamic Response of Substrate Phosphorylation to Kinase Modulation. Kinases Phosphatases. 2023;1(4):4. 10.3390/kinasesphosphatases1040015.

[17] Guo W, Hao B, Wang Q, Lu Y, Yue J. Requirement of B-Raf, C-Raf, and A-Raf for the growth and survival of mouse embryonic stem cells. Exp Cell Res. 2013;319(18):2801–11. 10.1016/j.yexcr.2013.09.006. 24051329

[18] Karna SKL, Lone BA, Ahmad F, Shahi N, Pokharel YR. Silencing of the CSNK2β gene by siRNA inhibits invasiveness and growth of MDA-MB-231 cells [Internet]. bioRxiv; 2018 [cited 2025 Jan 8]. p. 311092. Available from: https://www.biorxiv.org/content/ doi:10.1101/311092.

[19] Halloran D, Pandit V, Nohe A. The Role of Protein Kinase CK2 in Development and Disease Progression: A Critical Review. J Dev Biol. 2022;10(3):31. 10.3390/jdb10030031 . PubMed PMID: 35997395; PubMed Central PMCID: PMC9397010. PMC9397010 35997395

[20] Pinna LA. Protein kinase CK2. Int J Biochem Cell Biol. 1997;29(4):551–4. 10.1016/S1357-2725(96)00142-2. 9363631

[21] Azari M, Bahreini F, Uversky VN, Rezaei N. Current therapeutic approaches and promising perspectives of using bioengineered peptides in fighting chemoresistance in triple-negative breast cancer. Biochem Pharmacol. 2023;210:115459. 10.1016/j.bcp.2023.115459. 36813121

[22] Ferrer-Font L, Villamañan L, Arias-Ramos N, Vilardell J, Plana M, Ruzzene M, et al. Targeting Protein Kinase CK2: Evaluating CX-4945 Potential for GL261 Glioblastoma Therapy in Immunocompetent Mice. Pharmaceuticals (Basel). 2017;10(1):24. 10.3390/ph10010024 . PubMed PMID: 28208677; PubMed Central PMCID: PMC5374428. PMC5374428 28208677

[23] Al-Zoubi RM, Farhan A, Hanbali B, Al-Zoubi SR, Al-Qudimat AR, Al Zoubi MS, et al. Inhibiting CK2 in breast cancer: From molecular targets to drug candidates. Eur J Med Chem. 2026;308:118620. 10.1016/j.ejmech.2026.118620. 41759307

[24] Perea SE, Baladrón I, Valenzuela C, Perera Y. CIGB-300: A peptide-based drug that impairs the Protein Kinase CK2-mediated phosphorylation. Semin Oncol. 2018;45(1):58–67. 10.1053/j.seminoncol.2018.04.006. 30318085

[25] Perea SE, Baladron I, Garcia Y, Perera Y, Lopez A, Soriano JL, et al. CIGB-300, a synthetic peptide-based drug that targets the CK2 phosphoaceptor domain. Translational and clinical research. Mol Cell Biochem. 2011;356(1):45–50. 10.1007/s11010-011-0950-y. 21735096

[26] Wells CI, Drewry DH, Pickett JE, Tjaden A, Krämer A, Müller S, et al. Development of a potent and selective chemical probe for the pleiotropic kinase CK2. Cell Chem Biology. 2021;28(4):546–e55810. 10.1016/j.chembiol.2020.12.013. PMC8864761 33484635

[27] Bancet A, Frem R, Jeanneret F, Mularoni A, Bazelle P, Roelants C, et al. Cancer selective cell death induction by a bivalent CK2 inhibitor targeting the ATP site and the allosteric αD pocket. iScience. 2024;27(2):108903. 10.1016/j.isci.2024.108903. PMC10838953 38318383

[28] Ortín I, Ochoa-Callejero L, Werner C, Lindenblatt D, Niefind K, Martínez A, et al. Targeting Casein Kinase 2 and Histone Deacetylase with a Dual Inhibitor Effectively Reduces Tumor Growth in a Triple-Negative Breast Cancer Xenograft Model. ACS Pharmacol Transl Sci. 2025;8(7):2093–105. 10.1021/acsptsci.5c00192. PMC12260935 40672667

[29] Wińska P, Wielechowska M, Koronkiewicz M, Borowiecki P. Synthesis and Anticancer Activity of Novel Dual Inhibitors of Human Protein Kinases CK2 and PIM-1. Pharmaceutics. 2023;15(7):7. 10.3390/pharmaceutics15071991. PMC10385865 37514177

[30] Padgaonkar A, Cosenza S, Pallela V, Subbaiah DRCV, Reddy MR, Reddy EP. Abstract 4453: The dual CK2/TNIK inhibitor, ON108600 targets cancer stem cells and induces apoptosis of paclitaxel resistant triple-negative breast cancer cells. Cancer Res. 2015;75(15Supplement):4453. 10.1158/1538-7445.AM2015-4453.

[31] Sato K, Padgaonkar AA, Baker SJ, Cosenza SC, Rechkoblit O, Subbaiah DRCV, et al. Simultaneous CK2/TNIK/DYRK1 inhibition by 108600 suppresses triple negative breast cancer stem cells and chemotherapy-resistant disease. Nat Commun. 2021;12(1):4671. 10.1038/s41467-021-24878-z. PMC8333338 34344863

[32] Azari M, Bahreini F, Uversky VN, Rezaei N. Current therapeutic approaches and promising perspectives of using bioengineered peptides in fighting chemoresistance in triple-negative breast cancer. Biochem Pharmacol. 2023;210:115459. 10.1016/j.bcp.2023.115459 . PubMed PMID: 36813121. 36813121

[33] Nguyen HM, Paulishak W, Oladejo M, Wood L. Dynamic tumor microenvironment, molecular heterogeneity, and distinct immunologic portrait of triple-negative breast cancer: an impact on classification and treatment approaches. Breast Cancer. 2023;30(2):167–86. 10.1007/s12282-022-01415-4. 36399321

[34] Shu S, Lin CY, He HH, Witwicki RM, Tabassum DP, Roberts JM, et al. Response and resistance to BET bromodomain inhibitors in triple-negative breast cancer. Nature. 2016;529(7586):413–7. 10.1038/nature16508. PMC4854653 26735014

[35] Ossovskaya V, Wang Y, Budoff A, Xu Q, Lituev A, Potapova O, et al. Exploring Molecular Pathways of Triple-Negative Breast Cancer. Genes Cancer. 2011;2(9):870–9. doi:10.1177/1947601911432496 PubMed PMID: 22593799; PubMed Central PMCID: PMC3352156. 10.1177/1947601911432496 PMC3352156 22593799

[36] Yu K, Rohr J, Liu Y, Li M, Xu J, Wang K, et al. Progress in triple negative breast carcinoma pathophysiology: Potential therapeutic targets. Pathol - Res Pract. 2020;216(4):152874. 10.1016/j.prp.2020.152874. 32088086

[37] Kumar H, Gupta NV, Jain R, Madhunapantula SV, Babu CS, Kesharwani SS, et al. A review of biological targets and therapeutic approaches in the management of triple-negative breast cancer. J Adv Res. 2023;54:271–92. 10.1016/j.jare.2023.02.005. PMC10703733 36791960

[38] Fan Y, He S. The Characteristics of Tumor Microenvironment in Triple Negative Breast Cancer. CMAR. 2022;141–17. 10.2147/CMAR.S316700.<\/p> PMC8740624 35018117

[39] Nedeljković M, Damjanović A. Mechanisms of Chemotherapy Resistance in Triple-Negative Breast Cancer-How We Can Rise to the Challenge. Cells. 2019;8:(9):957. doi:10.3390/cells8090957 PubMed PMID: 31443516; PubMed Central PMCID: PMC6770896. 10.3390/cells8090957 PMC6770896 31443516

[40] Wu Y, Wang Y, Lin Y, Liu Y, Wang Y, Jia J, et al. Dub3 inhibition suppresses breast cancer invasion and metastasis by promoting Snail1 degradation. Nat Commun. 2017;8:14228. 10.1038/ncomms14228 . PubMed PMID: 28198361; PubMed Central PMCID: PMC5316870. PMC5316870 28198361

[41] Xu Y, Luo Y, Liang C. A regulation loop between Nrf1α and MRTF-A controls migration and invasion in MDA-MB-231 breast cancer cells. Int J Mol Med. 2018;42(5):2459. 10.3892/ijmm.2018.3816. PMC6192731 30106093

[42] Wu SY, Lee AY, Lai HT, Zhang H, Chiang CM. Phospho Switch Triggers Brd4 Chromatin Binding and Activator Recruitment for Gene-Specific Targeting. Mol Cell. 2013;49(5):843–57. 10.1016/j.molcel.2012.12.006. PMC3595396 23317504

[43] Serrano M. SHP2: a new target for pro-senescence cancer therapies. EMBO J. 2015;34(11):1439–41. 10.15252/embj.201591616. PMC4474521 25916826

[44] Sharifhoseini A, Heshmati M, Soltani A, Entezam M, Shirzad H, Sedehi M, et al. Effects of bromodomain and extra-terminal inhibitor JQ1 and interleukin-6 on breast cancer cells. Mol Biol Rep. 2023;50(10):8319–28. 10.1007/s11033-023-08718-5. 37589934

[45] Dowling CM, Hollinshead KER, Di Grande A, Pritchard J, Zhang H, Dillon ET, et al. Multiple screening approaches reveal HDAC6 as a novel regulator of glycolytic metabolism in triple-negative breast cancer. Sci Adv. 2021;7(3):eabc4897. 10.1126/sciadv.abc4897. PMC7810372 33523897

[46] Romieu-Mourez R, Landesman-Bollag E, Seldin DC, Traish AM, Mercurio F, Sonenshein GE. Roles of IKK Kinases and Protein Kinase CK2 in Activation of Nuclear Factor-κB in Breast Cancer1. Cancer Res. 2001;61(9):3810–8. 11325857

[47] Eddy SF, Guo S, Demicco EG, Romieu-Mourez R, Landesman-Bollag E, Seldin DC, et al. Inducible IκB Kinase/IκB Kinase ε Expression Is Induced by CK2 and Promotes Aberrant Nuclear Factor-κB Activation in Breast Cancer Cells. Cancer Res. 2005;65(24):11375–83. 10.1158/0008-5472.CAN-05-1602. 16357145

[48] Zhang D, Shi X, Zheng W, Zhang X, Chen Y. Rare HER2 L796P missense mutation promotes the growth and oncogenic signaling in breast cancer cells. Proteom – Clin Appl. 2024;18(1):2300061. 10.1002/prca.202300061. 37672800

[49] Drygin D, Ho CB, Omori M, Bliesath J, Proffitt C, Rice R, et al. Protein kinase CK2 modulates IL-6 expression in inflammatory breast cancer. Biochem Biophys Res Commun. 2011;415(1):163–7. 10.1016/j.bbrc.2011.10.046. 22027148

[50] Zecchin D, Moore C, Michailidis F, Horswell S, Rana S, Howell M, et al. Combined targeting of G protein-coupled receptor and EGF receptor signaling overcomes resistance to PI3K pathway inhibitors in PTEN‐null triple negative breast cancer. EMBO Mol Med. 2020;12(8):e11987. 10.15252/emmm.202011987. PMC7411640 32672423

[51] Lefebvre C, Pellizzari S, Bhat V, Jurcic K, Litchfield DW, Allan AL. Involvement of the AKT Pathway in Resistance to Erlotinib and Cabozantinib in Triple-Negative Breast Cancer Cell Lines. Biomedicines. 2023;11(9):9. 10.3390/biomedicines11092406. PMC10525382 37760847

[52] Tsuchiya Y, Taniguchi,Hiroaki I, Yoshiyuki, Morita, Tomoko, Karim M, Rezaul et al. Ohtake, Norihito,. The Casein Kinase 2-Nrf1 Axis Controls the Clearance of Ubiquitinated Proteins by Regulating Proteasome Gene Expression. Molecular and Cellular Biology. 2013;33(17):3461–72. 10.1128/MCB.01271-12 PubMed PMID: 23816881. 10.1128/MCB.01271-12 PMC3753846 23816881

[53] Gau D, Chawla P, Eder I, Roy P. Myocardin-related transcription factor’s interaction with serum-response factor is critical for outgrowth initiation, progression, and metastatic colonization of breast cancer cells. FASEB BioAdvances. 2022;4(8):509–23. 10.1096/fba.2021-00113. PMC9353439 35949508

[54] Meng C, He Y, Wei Z, Lu Y, Du F, Ou G, et al. MRTF-A mediates the activation of *COL1A1* expression stimulated by multiple signaling pathways in human breast cancer cells. Biomed Pharmacother. 2018;104:718–28. 10.1016/j.biopha.2018.05.092. 29807221

[55] Méndez O, Peg V, Salvans C, Pujals M, Fernández Y, Abasolo I, et al. Extracellular HMGA1 Promotes Tumor Invasion and Metastasis in Triple-Negative Breast Cancer. Clin Cancer Res. 2018;24(24):6367–82. 10.1158/1078-0432.CCR-18-0517. 30135148

[56] Balcioglu O, Gates BL, Freeman DW, Hagos BM, Mehrabad EM, Ayala-Talavera D, et al. Mcam stabilizes a luminal progenitor-like breast cancer cell state via Ck2 control and Src/Akt/Stat3 attenuation. npj Breast Cancer. 2024;10(1):1–13. 10.1038/s41523-024-00687-7. PMC11401886 39277578

[57] Cho YB, Ko IY, Kim HJ, Kim JW, Heo K, Na HJ, et al. Functional epitope mapping of cell surface glucose-regulated protein 94: A combinatorial approach for therapeutic targeting. Int J Biol Macromol. 2025;301:140374. 10.1016/j.ijbiomac.2025.140374. 39880265

[58] Kim HY, Kim YM, Hong S. CK2α-mediated phosphorylation of GRP94 facilitates the metastatic cascade in triple-negative breast cancer. Cell Death Discov. 2024;10(1):1–14. 10.1038/s41420-024-01956-x. PMC11035675 38649679

[59] Zheng W, Shen P, Yu C, Tang Y, Qian C, Yang C, et al. Ginsenoside Rh1, a novel casein kinase II subunit alpha (CK2α) inhibitor, retards metastasis via disrupting HHEX/CCL20 signaling cascade involved in tumor cell extravasation across endothelial barrier. Pharmacol Res. 2023;198:106986. 10.1016/j.phrs.2023.106986. 37944834

[60] Andrade D, Mehta M, Griffith J, Panneerselvam J, Srivastava A, Kim TD, et al. YAP1 inhibition radiosensitizes triple negative breast cancer cells by targeting the DNA damage response and cell survival pathways. Oncotarget. 2017;8(58):98495–508. 10.18632/oncotarget.21913. PMC5716745 29228705

[61] Huang L, Wen Y, Guo Q, Zhang C, Yang X, Li M, et al. CK2α-mediated phosphorylation of DUB3 promotes YAP1 stability and oncogenic functions. Cell Death Dis. 2025;16(1):1–14. 10.1038/s41419-024-07323-z. PMC11743126 39827153

[62] Pavitra E, Kancharla J, Gupta VK, Prasad K, Sung JY, Kim J, et al. The role of NF-κB in breast cancer initiation, growth, metastasis, and resistance to chemotherapy. Biomed Pharmacother. 2023;163:114822. 10.1016/j.biopha.2023.114822. 37146418

[63] Ho C, Rice R, Drygin D, Bliesath J, Streiner N, Siddiqui-Jain A, et al. CX-4945, a Selective and Orally Bioavailable Inhibitor of Protein Kinase CK2 Inhibits PI3K/Akt, JAK-STAT and NF-κB Signaling and Induces Apoptosis In Multiple Myeloma Cells. Blood. 2010;116(21):787. 10.1182/blood.V116.21.787.787.

[64] Shostak K, Chariot A. NF-κB, stem cells and breast cancer: the links get stronger. Breast Cancer Res. 2011;13(4):214. 10.1186/bcr2886. PMC3236328 21867572

[65] Wang M, Zhang Y, Xu Z, Qian P, Sun W, Wang X, et al. RelB sustains endocrine resistant malignancy: an insight of noncanonical NF-κB pathway into breast Cancer progression. Cell Commun Signal. 2020;18(1):128. 10.1186/s12964-020-00613-x. PMC7430126 32807176

[66] Manni S, Brancalion A, Mandato E, Tubi LQ, Colpo A, Pizzi M, et al. Protein Kinase CK2 Inhibition Down Modulates the NF-κB and STAT3 Survival Pathways, Enhances the Cellular Proteotoxic Stress and Synergistically Boosts the Cytotoxic Effect of Bortezomib on Multiple Myeloma and Mantle Cell Lymphoma Cells. PLoS ONE. 2013;8(9):e75280. 10.1371/journal.pone.0075280. PMC3785505 24086494

[67] Buontempo F, Orsini E, Lonetti A, Cappellini A, Chiarini F, Evangelisti C, et al. Synergistic cytotoxic effects of bortezomib and CK2 inhibitor CX4945 in acute lymphoblastic leukemia: turning off the prosurvival ER chaperone BIP/Grp78 and turning on the proapoptotic NFÎ^o^B. Oncotarget. 2015;7(2):1323–40. 10.18632/oncotarget.6361. PMC4811463 26593250

[68] Zhang Hping, Jiang R, yuan, Zhu Jyu, Sun K na, Huang Y, Zhou H, huan et al. PI3K/AKT/mTOR signaling pathway: an important driver and therapeutic target in triple-negative breast cancer. Breast Cancer. 2024;31(4):539–51. 10.1007/s12282-024-01567-5 PubMed PMID: 38630392; PubMed Central PMCID: PMC11194209. PMC11194209 38630392

[69] Tošić I, Frank DA. STAT3 as a mediator of oncogenic cellular metabolism: Pathogenic and therapeutic implications. Neoplasia. 2021;23(12):1167–78. 10.1016/j.neo.2021.10.003. PMC8569436 34731785

[70] Di Maira G, Brustolon F, Pinna LA, Ruzzene M. Dephosphorylation and inactivation of Akt/PKB is counteracted by protein kinase CK2 in HEK 293T cells. Cell Mol Life Sci. 2009;66(20):3363–73. 10.1007/s00018-009-0108-1. PMC11115639 19662498

[71] Di Maira G, Salvi M, Arrigoni G, Marin O, Sarno S, Brustolon F, et al. Protein kinase CK2 phosphorylates and upregulates Akt/PKB. Cell Death Differ. 2005;12(6):668–77. 10.1038/sj.cdd.4401604. 15818404

[72] Hassan A, Aubel C. The PI3K/Akt/mTOR Signaling Pathway in Triple-Negative Breast Cancer: A Resistance Pathway and a Prime Target for Targeted Therapies. Cancers. 2025;17(13):2232. 10.3390/cancers17132232. PMC12248877 40647529

[73] Dance M, Montagner A, Salles JP, Yart A, Raynal P. The molecular functions of Shp2 in the Ras/Mitogen-activated protein kinase (ERK1/2) pathway. Cell Signal. 2008;20(3):453–9. 10.1016/j.cellsig.2007.10.002. 17993263

[74] Grossmann KS, Rosário M, Birchmeier C, Birchmeier W. Chapter 2 - The Tyrosine Phosphatase Shp2 in Development and Cancer. In: Vande Woude GF, Klein G, editors. Advances in Cancer Research [Internet]. Academic Press; 2010 [cited 2026 Jun 11]. pp. 53–89. Available from: https://www.sciencedirect.com/science/article/pii/S0065230X10060021 10.1016/S0065-230X(10)06002-1 20399956

[75] Ahmed TA, Adamopoulos C, Karoulia Z, Wu X, Sachidanandam R, Aaronson SA, et al. SHP2 Drives Adaptive Resistance to ERK Signaling Inhibition in Molecularly Defined Subsets of ERK-Dependent Tumors. Cell Rep. 2019;26(1):65–e785. 10.1016/j.celrep.2018.12.013 . PubMed PMID: 30605687. PMC6396678 30605687

[76] Roskoski R. ERK1/2 MAP kinases: Structure, function, and regulation. Pharmacol Res. 2012;66(2):105–43. 10.1016/j.phrs.2012.04.005. 22569528

[77] Plotnikov A, Chuderland D, Karamansha Y, Livnah O, Seger R. Nuclear ERK Translocation is Mediated by Protein Kinase CK2 and Accelerated by Autophosphorylation | Cell Physiol Biochem. Cell Physiol Biochem. 2019;53(2):366–87. 10.33594/000000144 31385665

[78] Wang Z, Xu Q, Zhang N, Du X, Xu G, Yan X. CD146, from a melanoma cell adhesion molecule to a signaling receptor. Sig Transduct Target Ther. 2020;5(1):1–15. 10.1038/s41392-020-00259-8. PMC7421905 32782280

[79] Marzec M, Eletto D, Argon Y. GRP94: an HSP90-like protein specialized for protein folding and quality control in the Endoplasmic Reticulum. Biochim Biophys Acta. 2012;1823(3):774–87. 10.1016/j.bbamcr.2011.10.013 . PubMed PMID: 22079671; PubMed Central PMCID: PMC3443595. PMC3443595 22079671

[80] Duan X, Iwanowycz S, Ngoi S, Hill M, Zhao Q, Liu B. Molecular Chaperone GRP94/GP96 in Cancers: Oncogenesis and Therapeutic Target. Front Oncol. 2021;11. 10.3389/fonc.2021.629846. PMC8062746 33898309

[81] Schutyser E, Struyf S, Van Damme J. The CC chemokine CCL20 and its receptor CCR6. Cytokine Growth Factor Rev. 2003;14(5):409–26. 10.1016/s1359-6101. (03)00049-2 PubMed PMID: 12948524. 12948524

[82] Chen W, Qin Y, Wang D, Zhou L, Liu Y, Chen S, et al. CCL20 triggered by chemotherapy hinders the therapeutic efficacy of breast cancer. PLoS Biol. 2018;16(7):e2005869. 10.1371/journal.pbio.2005869. PMC6082578 30052635

[83] Zhou JX, Agborbesong E, Li LX, Li X. Bromodomain Protein BRD4-Mediated Mutant p53 Transcription Promotes TNBC Progression. Int J Mol Sci. 2022;23(23):23. 10.3390/ijms232315163. PMC9738555 36499487

[84] Zhang X, Ozawa Y, Lee H, Wen YD, Tan TH, Wadzinski BE, et al. Histone deacetylase 3 (HDAC3) activity is regulated by interaction with protein serine/threonine phosphatase 4. Genes Dev. 2005;19(7):827–39. 10.1101/gad.1286005. PMC1074320 15805470

[85] Watabe M, Nakaki T. Protein kinase CK2 regulates the formation and clearance of aggresomes in response to stress. J Cell Sci. 2011;124(9):1519–32. 10.1242/jcs.081778. 21486957

[86] Tangutoori S, Baldwin P, Sridhar S. PARP inhibitors: A new era of targeted therapy. Maturitas. 2015;81(1):5–9. 10.1016/j.maturitas.2015.01.015. 25708226

[87] Kanev PB, Atemin A, Stoynov S, Aleksandrov R. PARP1 roles in DNA repair and DNA replication: The basi(c)s of PARP inhibitor efficacy and resistance. Semin Oncol. 2024;PARP Inhibitors51(1):2–18. 10.1053/j.seminoncol.2023.08.001. 37714792

[88] Yu WH. Development Of Rational Combination Therapy With Parp Inhibitors And Kinase Inhibitors In Tnbc. Dissertations & Theses (Open Access) [Internet]. 2016 Aug 1. Available from: https://digitalcommons.library.tmc.edu/utgsbs_dissertations/682

[89] Wang C, Tian L, He Q, Lin S, Wu Y, Qiao Y, et al. Targeting CK2-mediated phosphorylation of p53R2 sensitizes BRCA-proficient cancer cells to PARP inhibitors. Oncogene. 2023;42(40):2971–84. 10.1038/s41388-023-02812-5. 37620447

[90] Guo Q, Zhao J, Li Y, Zhang C, Shen X, Liu L, et al. CK2–HTATSF1–TOPBP1 signaling axis modulates tumor chemotherapy response. J Biol Chem. 2024;300(6). 10.1016/j.jbc.2024.107377 . PubMed PMID: 38762174. PMC11208909 38762174

[91] Sinha S, Sharma S, Vora J, Shrivastava N. Emerging role of sirtuins in breast cancer metastasis and multidrug resistance: Implication for novel therapeutic strategies targeting sirtuins. Pharmacol Res. 2020;158:104880. 10.1016/j.phrs.2020.104880. 32442721

[92] Bae JS, Park SH, Jamiyandorj U, Kim KM, Noh SJ, Kim JR, et al. CK2α/CSNK2A1 Phosphorylates SIRT6 and Is Involved in the Progression of Breast Carcinoma and Predicts Shorter Survival of Diagnosed Patients. Am J Pathol. 2016;186(12):3297–315. 10.1016/j.ajpath.2016.08.007. 27746184

[93] Kang H, Jung JW, Kim MK, Chung JH. CK2 Is the Regulator of SIRT1 Substrate-Binding Affinity, Deacetylase Activity and Cellular Response to DNA-Damage. PLoS ONE. 2009;4(8):e6611. 10.1371/journal.pone.0006611. PMC2721681 19680552

[94] Hatakeyama M, Weinberg RA. The role of RB in cell cycle control. Prog Cell Cycle Res. 1995;1:9–19. 10.1007/978-1-4615-1809-9_2. 1809-9_2 PubMed PMID: 9552350. 9552350

[95] Knudsen ES, Knudsen KE. Retinoblastoma tumor suppressor: where cancer meets the cell cycle. Exp Biol Med (Maywood). 2006;231(7):1271–81. doi:10.1177/153537020623100713 PubMed PMID: 16816134. 10.1177/153537020623100713 16816134

[96] Burkhart DL, Sage J. Cellular mechanisms of tumour suppression by the retinoblastoma gene. Nat Rev Cancer. 2008;8(9):671–82. doi:10.1038/nrc2399 PubMed PMID: 18650841; PubMed Central PMCID: PMC6996492. 10.1038/nrc2399 PMC6996492 18650841

[97] Bulanova D, Akimov Y, Senkowski W, Oikkonen J, Gall-Mas L, Timonen S, et al. A synthetic lethal dependency on casein kinase 2 in response to replication-perturbing therapeutics in RB1-deficient cancer cells. Sci Adv. 2024;10(21):eadj1564. 10.1126/sciadv.adj1564 . PubMed PMID: 38781347; PubMed Central PMCID: PMC11114247. PMC11114247 38781347

[98] Gottardo MF, Capobianco CS, Sidabra JE, Garona J, Perera Y, Perea SE, et al. Preclinical efficacy of CIGB-300, an anti-CK2 peptide, on breast cancer metastasic colonization. Sci Rep. 2020;10(1):14689. 10.1038/s41598-020-71854-6. PMC7477577 32895446

[99] Karna SKL, Lone BA, Ahmad F, Shahi N, Pokharel YR. Knockdown of CSNK2β suppresses MDA-MB231 cell growth, induces apoptosis, inhibits migration and invasion. EXCLI J. 2020;19:1211–26. 10.17179/excli2020-2363. PMC7527516 33013272

[100] Zhou Y, Shen JK, Hornicek FJ, Kan Q, Duan Z. The emerging roles and therapeutic potential of cyclin-dependent kinase 11 (CDK11) in human cancer. Oncotarget. 2016;7(26):40846–59. 10.18632/oncotarget.8519. PMC5130049 27049727

[101] Kren BT, Unger GM, Abedin MJ, Vogel RI, Henzler CM, Ahmed K, et al. Preclinical evaluation of cyclin dependent kinase 11 and casein kinase 2 survival kinases as RNA interference targets for triple negative breast cancer therapy. Breast Cancer Res. 2015;17(1):19. 10.1186/s13058-015-0524-0. PMC4344788 25837326

[102] Sato K, Baker SJ, Reddy EP, Irie HY. Abstract PD3-08: Novel cancer stem cell inhibitor 108600 modulates tumor immunomicroenvironment of triple negative breast cancer (TNBC). Cancer Res. 2022;82(4Supplement):PD3–08. 10.1158/1538-7445.SABCS21-PD3-08.

[103] Kaur S, Reginauld B, Razjooyan S, Phi T, Singh SP, Meyer TJ, et al. Effects of a humanized CD47 antibody and recombinant SIRPα proteins on triple negative breast carcinoma stem cells. Front Cell Dev Biol. 2024;12. 10.3389/fcell.2024.1356421. PMC10940465 38495618

[104] Leis O, Eguiara A, Lopez-Arribillaga E, Alberdi MJ, Hernandez-Garcia S, Elorriaga K, et al. Sox2 expression in breast tumours and activation in breast cancer stem cells. Oncogene. 2012;31(11):1354–65. 10.1038/onc.2011.338. 21822303

[105] Mukherjee P, Gupta A, Chattopadhyay D, Chatterji U. Modulation of SOX2 expression delineates an end-point for paclitaxel-effectiveness in breast cancer stem cells. Sci Rep. 2017;7(1):9170. 10.1038/s41598-017-08971-2. PMC5569040 28835684

[106] Gwak JM, Kim M, Kim HJ, Jang MH, Park SY. Expression of embryonal stem cell transcription factors in breast cancer: Oct4 as an indicator for poor clinical outcome and tamoxifen resistance. Oncotarget. 2017;8(22):36305–18. 10.18632/oncotarget.16750. PMC5482656 28422735

[107] Fultang N, Chakraborty M, Peethambaran B. Regulation of cancer stem cells in triple negative breast cancer. cdr. 2021;4(2):321–42. 10.20517/cdr.2020.106. PMC9019272 35582030

[108] Nitta RT, Gholamin S, Feroze AH, Agarwal M, Cheshier SH, Mitra SS, et al. Casein kinase 2α regulates glioblastoma brain tumor-initiating cell growth through the β-catenin pathway. Oncogene. 2015;34(28):3688–99. 10.1038/onc.2014.299. PMC4369469 25241897

[109] Silva-Pavez E, Tapia JC. Protein Kinase CK2 in Cancer Energetics. Front Oncol. 2020;10. 10.3389/fonc.2020.00893. PMC7315807 32626654

[110] Gray GK, McFarland BC, Rowse AL, Gibson SA, Benveniste EN. Therapeutic CK2 inhibition attenuates diverse prosurvival signaling cascades and decreases cell viability in human breast cancer cells. Oncotarget. 2014;5(15):6484–96. 10.18632/oncotarget.2248 . PubMed PMID: 25153725; PubMed Central PMCID: PMC4171645. PMC4171645 25153725

[111] Yang J, Liao D, Chen C, Liu Y, Chuang TH, Xiang R, et al. Tumor-Associated Macrophages Regulate Murine Breast Cancer Stem Cells Through a Novel Paracrine EGFR/Stat3/Sox-2 Signaling Pathway. Stem Cells. 2013;31(2):248–58. 10.1002/stem.1281. 23169551

[112] Shkoda A, Town JA, Griese J, Romio M, Sarioglu H, Knöfel T, et al. The Germinal Center Kinase TNIK Is Required for Canonical NF-κB and JNK Signaling in B-Cells by the EBV Oncoprotein LMP1 and the CD40 Receptor. PLoS Biol. 2012;10(8):e1001376. 10.1371/journal.pbio.1001376. PMC3419181 22904686

[113] Laham AJ, El-Awady R, Saber-Ayad M, Wang N, Yan G, Boudreault J, et al. Targeting the DYRK1A kinase prevents cancer progression and metastasis and promotes cancer cells response to G1/S targeting chemotherapy drugs. npj Precis Onc. 2024;8(1):1–14. 10.1038/s41698-024-00614-w. PMC11153725 38839871

[114] Zhao X, Wei Y, Chu YY, Li Y, Hsu JM, Jiang Z, et al. Phosphorylation and Stabilization of PD-L1 by CK2 Suppresses Dendritic Cell Function. Cancer Res. 2022;82(11):2185–95. 10.1158/0008-5472.CAN-21-2300. PMC11957750 35385574

[115] Han Y, Liu D, Li L. PD-1/PD-L1 pathway: current researches in cancer. PMC7136921 32266087

[116] Tang Q, Chen Y, Li X, Long S, Shi Y, Yu Y, et al. The role of PD-1/PD-L1 and application of immune-checkpoint inhibitors in human cancers. Front Immunol. 2022;13. 10.3389/fimmu.2022.964442. PMC9513184 36177034

[117] PGRMC1 promotes triple-negative breast cancer cell growth via suppressing ferroptosis: Climacteric. Vol 26, No 2 - Get Access. Climacteric [Internet]. [cited 2026 Jun 7]. Available from: https://www.tandfonline.com/doi/full/10.1080/13697137.2023.2170225 36724820

[118] Tabnak P, Ebrahimnezhad M, HajiEsmailPoor Z. Genetic mutations governing ferroptosis sensitivity and resistance: a precision approach to cancer therapy. Cell Death Dis 2026 May 7. 10.1038/s41419-026-08796-w PMC13315318 42098074

[119] Taylor KM, Hiscox S, Nicholson RI, Hogstrand C, Kille P. Protein Kinase CK2 Triggers Cytosolic Zinc Signaling Pathways by Phosphorylation of Zinc Channel ZIP7. Sci Signal. 2012;5(210):ra11–11. 10.1126/scisignal.2002585. PMC3428905 22317921

[120] Zaman MS, Johnson AJ, Petersingham G, Muench GW, Dong Q, Wu MJ. Protein kinase CK2 is involved in zinc homeostasis in breast and prostate cancer cells. Biometals. 2019;32(6):861–73. 10.1007/s10534-019-00218-z. 31583500

[121] Chojnowski JE, Li R, Tsang T, Alfaran FH, Dick A, Cocklin S, et al. Copper Modulates the Catalytic Activity of Protein Kinase CK2. Front Mol Biosci. 2022;9. 10.3389/fmolb.2022.878652. PMC9224766 35755824

[122] Focaccio A, Rossi L, De Luca A. A spotlight on the role of copper in the epithelial to mesenchymal transition. Life Sci. 2024;354:122972. 10.1016/j.lfs.2024.122972. 39142503

[123] Ryan C. Silmitasertib Drives Disease Control in Advanced Basal Cell Carcinoma | OncLive [Internet]. 2026 [cited 2026 Jun 8]. Available from: https://www.onclive.com/view/silmitasertib-drives-disease-control-in-advanced-basal-cell-carcinoma

[124] Sarduy MR, García I, Coca MA, Perera A, Torres LA, Valenzuela CM, et al. Optimizing CIGB-300 intralesional delivery in locally advanced cervical cancer. Br J Cancer. 2015;112(10):1636–43. 10.1038/bjc.2015.137. PMC4430720 25880012

[125] Silva-Pavez E, Villar P, Trigo C, Caamaño E, Niechi I, Pérez P, et al. CK2 inhibition with silmitasertib promotes methuosis-like cell death associated to catastrophic massive vacuolization of colorectal cancer cells. Cell Death Dis. 2019;10(2):73. 10.1038/s41419-019-1306-x. PMC6347595 30683840

[126] Borad MJ, Bai LY, Richards D, Mody K, Hubbard J, Rha SY, et al. Silmitasertib plus gemcitabine and cisplatin first-line therapy in locally advanced/metastatic cholangiocarcinoma: A Phase 1b/2 study. Hepatology. 2023;77(3):760. 10.1002/hep.32804. 36152015

[127] Al-Qadhi MA, Yahya TAA, El-Nassan HB. Recent Advances in the Discovery of CK2 Inhibitors. ACS Omega. 2024;9(19):20702–19. 10.1021/acsomega.3c10478. PMC11097362 38764653

[128] Franchin C, Borgo C, Zaramella S, Cesaro L, Arrigoni G, Salvi M, et al. Exploring the CK2 Paradox: Restless. Danger Dispensable Pharmaceuticals. 2017;10(1):11. 10.3390/ph10010011. PMC5374415 28117670

[129] Wiranowska M. Advances in the use of chitosan and chlorotoxin- functionalized chitosan polymers in drug delivery and detection of glioma – A review. Carbohydr Polym Technol Appl. 2024;7:100427. 10.1016/j.carpta.2024.100427.

[130] Chou ST, Patil R, Galstyan A, Gangalum PR, Cavenee WK, Furnari FB, et al. Simultaneous blockade of interacting CK2 and EGFR pathways by tumor-targeting nanobioconjugates increases therapeutic efficacy against glioblastoma multiforme. J Controlled Release. 2016;244:14–23. 10.1016/j.jconrel.2016.11.001. PMC5308909 27825958

[131] Martínez R, Di Geronimo B, Pastor M, Zapico JM, Coderch C, Panchuk R, et al. Multitarget Anticancer Agents Based on Histone Deacetylase and Protein Kinase CK2 Inhibitors. Molecules. 2020;25(7):7. 10.3390/molecules25071497. PMC7180456 32218358

[132] Liu T, Wan Y, Xiao Y, Xia C, Duan G. Dual-Target Inhibitors Based on HDACs: Novel Antitumor Agents for Cancer Therapy. J Med Chem. 2020;63(17):8977–9002. 10.1021/acs.jmedchem.0c00491. 32320239

